# Development and function of chicken XCR1^+^ conventional dendritic cells

**DOI:** 10.3389/fimmu.2023.1273661

**Published:** 2023-10-25

**Authors:** Zhiguang Wu, Barbara Shih, Joni Macdonald, Dominique Meunier, Kris Hogan, Cosmin Chintoan-Uta, Hazel Gilhooley, Tuanjun Hu, Mariana Beltran, Neil C. Henderson, Helen M. Sang, Mark P. Stevens, Michael J. McGrew, Adam Balic

**Affiliations:** ^1^ The Roslin Institute, University of Edinburgh, Midlothian, United Kingdom; ^2^ Division of Biomedical and Life Sciences, Faculty of Health and Medicine, Lancaster University, Lancaster, United Kingdom; ^3^ Centre for Inflammation Research, The Queen’s Medical Research Institute, University of Edinburgh, Edinburgh, United Kingdom; ^4^ Medical Research Council (MRC) Human Genetics Unit, Institute of Genetics and Cancer, University of Edinburgh, Edinburgh, United Kingdom; ^5^ Department of Biochemistry and Pharmacology, Bio21 Molecular Science and Biotechnology Institute, The University of Melbourne, Parkville, VIC, Australia

**Keywords:** chicken, conventional dendritic cells, XCR1, conditional ablation, single cell RNA-seq

## Abstract

Conventional dendritic cells (cDCs) are antigen-presenting cells (APCs) that play a central role in linking innate and adaptive immunity. cDCs have been well described in a number of different mammalian species, but remain poorly characterised in the chicken. In this study, we use previously described chicken cDC specific reagents, a novel gene-edited chicken line and single-cell RNA sequencing (scRNAseq) to characterise chicken splenic cDCs. In contrast to mammals, scRNAseq analysis indicates that the chicken spleen contains a single, chemokine receptor XCR1 expressing, cDC subset. By sexual maturity the XCR1^+^ cDC population is the most abundant mononuclear phagocyte cell subset in the chicken spleen. scRNAseq analysis revealed substantial heterogeneity within the chicken splenic XCR1^+^ cDC population. Immature MHC class II (MHCII)^LOW^ XCR1^+^ cDCs expressed a range of viral resistance genes. Maturation to MHCII^HIGH^ XCR1^+^ cDCs was associated with reduced expression of anti-viral gene expression and increased expression of genes related to antigen presentation via the MHCII and cross-presentation pathways. To visualise and transiently ablate chicken XCR1^+^ cDCs *in situ*, we generated *XCR1*-iCaspase9-RFP chickens using a CRISPR-Cas9 knockin transgenesis approach to precisely edit the *XCR1* locus, replacing the XCR1 coding region with genes for a fluorescent protein (TagRFP), and inducible Caspase 9. After inducible ablation, the chicken spleen is initially repopulated by immature CD1.1^+^ XCR1^+^ cDCs. XCR1^+^ cDCs are abundant in the splenic red pulp, in close association with CD8^+^ T-cells. Knockout of XCR1 prevented this clustering of cDCs with CD8^+^ T-cells. Taken together these data indicate a conserved role for chicken and mammalian XCR1^+^ cDCs in driving CD8^+^ T-cells responses.

## Introduction

According to the Food & Agriculture Organisation, in 2021 an estimated 74 billion broiler chickens were killed for meat and laying hens produced 1.6 trillion eggs (1). Production of poultry on this scale is greatly facilitated by vaccination. However, a lack of knowledge of the sites, mechanisms and cell types involved in antigen presentation in the chicken hampers the development of new, more effective vaccines.

Conventional dendritic cells (cDCs) are potent activators of adaptive immune responses due to their ability to efficiently capture, process and present antigen to naïve T cells and drive clonal expansion of antigen-specific T-cell responses (2–5). The development and implementation of novel avian vaccines will require new knowledge of chicken cDC biology. In mammals, cDCs are rare Flt3 (CD135)-expressing cells (6) comprised of two functionally specialised subsets (5, 7). Despite emerging evidence for functional plasticity (8), the generation of distinct immune responses has been attributed to specific cDC subsets. Mammalian XCR1^+^ cDCs (cDC1) are described as being specialised for the induction of Th1 immune responses and the presentation of exogenously derived antigens to CD8^+^ T-cells via the MHC class I (MHCI) pathway (a process known as “cross-presentation”) (9–13). By contrast, the XCR1^-^ mammalian cDC2 subset participates in the induction of Th2 and Th17 immune responses (14, 15).

Transcriptomic approaches have identified a chicken immune cell population expressing genes associated with the mammalian cDC1 subset (including *XCR1, FLT3, ZBTB46, ID2, IRF8, CADM1*) in the chicken spleen, liver and lungs (16–19). More recently, we developed tools to specifically identify and characterise chicken cDCs (20). In agreement with earlier transcriptomic approaches, we demonstrated that chickens contain a single XCR1^+^ cDC population that appears to be the counterpart of the mammalian cDC1 subset (18, 20). However, chicken splenic XCR1^+^ cDC showed significant differences to the mammalian cDC1 subset in terms of relative abundance in the spleen and liver, the expression of high levels of CSF1R and lack CSF2R expression (20). It remains unclear if the processes regulating the development of chicken splenic XCR1^+^ cDCs are conserved with mammals, nor if they have the same functional specialisations as reported for the mammalian XCR1^+^ cDC1 subset.

The vertebrate spleen comprises of two main types of tissue, white pulp (WP) and red pulp (RP). Splenic RP is rich in red blood cells whereas the WP is densely packed with immune cells (21). The afferent splenic artery branches into the central artery (CA), which further divides into penicillar capillaries (21). Splenic microstructure has best been described in mice which differs significantly from that of many other vertebrates, including humans and chickens (21). In mice, the CA is surrounded by WP composed of successive layers of immune cells, a T-cell rich structure known as the periarteriolar lymphoid sheath (PALS), followed by B-cell follicles, and finally the marginal zone (MZ) which marks the boundary between the WP and RP (22). Blood borne antigens enter RP, or the marginal sinuses via the CA where they encounter specialised MZ macrophage and B-cell subsets (21, 22). In chickens (23) and humans (21) large accumulations of B-cells and macrophages surround the penicillar capillaries. In chickens, the penicillar capillaries are fenestrated enabling blood-borne antigens, cells and pathogens access to splenic immune cells via the penicillar capillaries (23).

Murine cDC1s are found in both RP and WP area (24–26). During infection with mouse cytomegalovirus (MCMV), chemokine (C motif) ligand (XCL1) producing natural killer cells (NKs) attract and activate cDC1 in the RP resulting in their relocation to the PALS T-cell zone in the WP where they interact with CD8^+^ T-cells (26). In contrast to the murine spleen, chicken CD8^+^ T-cells are mainly located in the RP (27), not the T-cell zone of the PALS, which is largely composed of CD4^+^ T-cells (27). It remains unclear where XCR1^+^ cDCs reside in the chicken spleen.

Here we investigated the development, diversity and regulation of XCR1^+^ cDCs in the chicken spleen. Using flow cytometry and scRNAseq analysis we show that chicken splenic cDCs consist of a single, but heterogeneous, population of XCR1^+^ cells. Chicken XCR1^+^ cDCs show gene expression consistent with a specialisation for the recognition of viral pathogens and for antigen presentation via the MHCII- and MHCI-dependent cross-presentation pathways. We developed a novel *XCR1*-iCaspase9-TagRFP gene-edited chicken line that enables visualisation and conditional ablation of XCR1^+^ cDCs. Our findings demonstrate that assumptions on what constitutes the conserved features of XCR1^+^ cDCs in mammals do not fully apply in chickens, and highlights the requirement to develop avian specific tools to gain further knowledge of the avian immune system for the improvement of vaccine-mediated immunity.

## Materials and methods

### Chickens and welfare

All birds were obtained from the National Avian Research Facility (NARF) at The Roslin Institute, University of Edinburgh. All birds were hatched and housed in premises licensed under a UK Home Office Establishment License in full compliance with the Animals (Scientific Procedures) Act 1986 and the Code of Practice for Housing and Care of Animals Bred, Supplied or Used for Scientific Purposes. *CSF1R*-eGFP transgenic chickens (28), from newly hatched chicks to 20 weeks of age, were used for initial analysis of XCR1^+^ cDCs. Production of founder birds, the *XCR1*-reporter line and the *XCR1* knockout line was carried out under UK Home Office Licenses (70/8528; 70/8940 and PP9565661). Inducible ablation of *XCR1*-iCaspase9-RFP^+^ cDCs by B/B homodimerizer drug was carried out under UK Home Office Licenses (PCD70CB48 and PP3522089). Experimental protocols and studies were approved by the Roslin Institute Animal Welfare and Ethical Review Board.

### Tissue processing for flow cytometric analysis

Chicken splenocytes were isolated from birds as described previously (20). To isolate the peripheral blood mononuclear cells (PBMCs), 2-5 ml blood was collected into Eppendorf tubes containing 50 μl of 0.5M EDTA (Sigma-Aldrich). Blood was diluted in phosphate-buffered saline (PBS) at a 1:1 ratio, layered on Histopaque (1077-1, Sigma-Aldrich) and spun at 400 × g for 30 min without braking. Mononuclear cells were collected from the gradient interface and washed twice with 1.0% bovine serum albumin (BSA, Sigma-Aldrich) in PBS (BSA/PBS).

To isolate bone marrow cells, femurs and tibias were flushed with PBS and the cells disaggregated by forcing through a 100 μm nylon cell strainer (Corning Inc.). Cells were spun at 500 × g for 10 min. To remove red blood cells, the cell pellet was re-suspended in an appropriate volume of PBS and carefully layered on Histopaque (1077-1, Sigma-Aldrich) and spun at 400 × g for 30 min without braking. Cells were collected from the gradient interface and washed 2 times with PBS/BSA.

To isolate cells from the skin, after removing fat and feathers, the skin was cut into small pieces (2.5 cm^2^) and digested with 2.5 mg/ml Dispase (Roche) while incubating in a 37°C water bath for 1 h with brief vortexing every 10 minutes. Remaining adipose and subcutaneous tissues were removed under a dissecting microscope and the epidermis/dermis layer was digested with 1 mg/ml Collagenase/Dispase/0.1 mg/ml DNase (Roche) in 5 ml of Hanks’ Balanced Salt Solution (HBSS, Thermo Fisher Scientific) with pulse vortexing and incubation in 37°C water bath for another 1-1.5 h. The skin samples were then minced in a petri dish using two scalpels and the resulting cell suspension passed through 100 μm strainer. Cells were then washed with 10 ml PBS/BSA.

To isolate immune cells from Ileum and Peyer’s Patches, the small intestine from *CSF1R*-eGFP transgenic birds was initially flushed with PBS to remove remaining intestinal content. Tissues were then dissected under a fluorescence microscope to isolate ileal Peyer’s patch from the non-lymphoid ileum (28). Tissue samples were cut into 2.5 cm^2^ pieces and washed three times in 50 ml Falcon conical tubes with 20 ml of complete media [CM, HBSS with 2% heat-inactivated fetal bovine serum (FBS, Sigma-Aldrich)] by vortexing for 30 s and then replacing the CM. To remove the mucus, 25 ml of CM containing 1 mM dithiothreitol (DTT, Thermo Fisher Scientific) was added to the tissue samples in 50 ml Falcon tubes which were incubated in a shaking incubator at 37°C and 220 rpm for 20 min, followed by vortexing for 30 s. To remove epithelial cells, tissue samples were incubated in 50 ml Falcon tubes with 25 ml of CM/EDTA (CM with 1.3 mM EDTA) at 37°C in a shaking incubator at 220 rpm for 40 min, followed by vortexing for 30 s. Tissue samples were then rinsed with CM and then digested and homogenised using a Potter-Elvehjem Polytetrafluoroethylene (PTFE) pestle and glass mortar as described for isolation of cDC from spleen (20).

### Flow cytometry analysis

Single-cell suspensions from tissues or blood were prepared and resuspended in cold FACS buffer (PBS, 1.0% BSA (w/v) and 0.05% sodium azide (w/v); Sigma-Aldrich) and placed on ice for 10 min. Cells were then incubated with reagents listed in **Table 1** in FACS buffer for 30 min on ice in the dark. If required, cells were washed and incubated with secondary antibodies for 20 min on ice in the dark. Cells were then washed three times, resuspended in cold FACS buffer and stained with SYTOX^®^ Blue Dead Cell Stain (Invitrogen; 1.0mM stock, 1/4000 dilution) prior to analysis with a LRSFortessa flow cytometer (BD Biosciences). Data were analysed using FlowJo V10 software. Dead cells were excluded by SYTOX^®^ Blue staining and doublets were then discriminated based on signal processing (FSC-A/W). Fluorescence minus one controls (FMO) were used to confirm gating strategies.

**Table 1:** Reagents used for flow cytometry and immunofluorescent microscopy analysis.

Reagent/Clone	Target	Isotype	Conjugate or secondary antibody	Source/Reference
Mouse anti-chicken CD45/clone LT-40	CD45	Mouse IgM	R-phycoerythrin (R-PE) or Alexa Fluor®(AF) 647	Southern Biotech
Mouse anti-chicken CD45/ clone AV53	CD45	Mouse IgG1	Rat Anti-mouse IgG1-Brilliant Violet™ (BV) 711 (A85-1; BD Biosciences)	Institute for Animal Health, UK
Mouse anti-Bu-1/clone AV20	Bu-1a/b	Mouse IgG1	R-PE, AF647 or Donkey anti-mouse IgG-AF594	Southern Biotech
Mouse anti-CD3/clone CT-3	CD3	Mouse IgG1	R-PE, AF647 or Donkey anti-mouse IgG-AF594	Southern Biotech
Mouse anti-chicken Monocytes/Macrophages /clone KUL01	MRC1L-B	Mouse IgG1	R-PE or AF647 or Rat Anti-mouse IgG1-BV711 (A85-1, BD Biosciences)	Southern Biotech
Mouse anti-chicken MHC II /clone 2G11	MHC class II	Mouse IgG1	R-PE or AF647	Southern Biotech
Mouse anti-chicken CD4/clone CT-4	CD4	Mouse IgG1	Rat Anti-mouse IgG1-BV711 (A85-1, BD Biosciences)	Southern Biotech
Mouse anti-chicken CD8β /clone EP42	CD8β	Mouse IgG2a	Goat anti-mouse IgG2a-AF647 (Invitrogen)	Southern Biotech
Mouse anti-chicken CD1.1/clone CB3	CD1.1	Mouse IgG1	Streptavidin-BV711 (BD Biosciences)	Southern Biotech
Mouse anti-chicken FLT3/clone ROS-AV184	FLT3	Mouse IgG1	AF647	Roslin Institute (20)
XCL1 peptide	XCR1	N/A	AF647	Roslin Institute (20)
RFP Antibody (710530)	Red fluorescent protein	Rabbit polyclonal	Donkey anti-Rabbit IgG-AF647	ThermoFisher Scientific, UK
GFP Antibody (600-101-215M)	Green fluorescent protein	Goat polyclonal	Donkey anti-Goat IgG-AF488	ThermoFisher Scientific, UK
CVI-ChNL-68.1	Unknown antigen; reported to stain chicken monocytes, macrophages, and interdigitating cells	Mouse IgG1	Donkey anti-mouse IgG-AF594	Prionics Lelystad B.V., Netherlands
CVI-ChNL-74.2	Unknown antigen; reported to stain chicken macrophage subsets	Mouse IgG1	Donkey anti-mouse IgG-AF594	Prionics Lelystad B.V., Netherlands
Mouse anti-chicken BAFF-R/clone 2C4	BAFFR	Mouse IgG1	Donkey anti-mouse IgG-AF594	Bio-Rad Antibodies, UK

N/A, not applicable.

For DNA content staining, splenocytes were counted, washed with PBS and stained with Zombie Violet™ Fixable dye (Biolegend) at 1/500 in PBS at room temperature (RT) for 15 min in the dark. Cells were then washed twice with FACS buffer (with 1.0% (w/v) BSA) and stained with synthetic chicken XCL1 peptide conjugated to Alexa Fluor 647 (XCL1^AF647^) for 30 min on ice. Cells were then washed twice with PBS and fixed with 2.0% paraformaldehyde (PFA, Sigma-Aldrich) at RT for 20 min. Cells were further washed twice with PBS and fixed with ice-cold 70% ethanol for 24 h. Cells were then washed twice with PBS and stained with 0.5 ml of FxCycle™ PI/RNase Staining Solution (Invitrogen™ F10797) per sample. Samples were incubated in the dark for 15–30 min prior to analysis using a BD LRSFortessa.

### Immunofluorescent staining and confocal imaging of tissue sections

Tissue samples were trimmed into 1.0 cm^2^ blocks and fixed overnight at 4°C in 4% PFA/PBS. Samples were removed from PFA/PBS and placed in 10% sucrose (w/v; Sigma-Aldrich)/PBS at 4°C overnight. Samples were then placed in 15/20/25/30% sucrose/PBS (w/v) for 24 h at 4°C for each sucrose concentration. Tissue samples were embedded in Cellpath™ OCT embedding matrix (Fisher Scientific UK Ltd, Loughborough, UK) and snap-frozen at −80˚C for two hours. 10µm sections were cut onto Superfrost Plus slides (Menzel-Gläser, Braunschweig, Germany) and air-dried for 1 h at RT. All primary antibodies used in this study are shown in **Table 1**. All slides were blocked for one hour in 10% normal horse serum (Sigma-Aldrich), 0.1% Triton X-100 (Sigma-Aldrich) in PBS (HST-PBS). All primary antibodies were diluted in blocking reagent (above) and incubated at 4°C overnight, washed for 20 min in PBS, followed by incubation with secondary antibodies for two hours (donkey anti-mouse IgG Alexa Fluor 594, donkey anti-mouse IgG1 Alexa Fluor 594, donkey anti-mouse IgG2a Alexa Fluor 647; Thermo Fisher Scientific) used at 1/300 dilution and mounted in ProLong^®^ Gold Antifade Mountant (Thermo Fisher Scientific). Where appropriate, sections were counterstained with 1 μg/ml 4′, 6′-diamidino-2-phenylindole (DAPI; Sigma-Aldrich) in the final incubation step. Samples were imaged using an inverted confocal microscope (Zeiss LSM710) and images were analysed using Zeiss ZEN 3·1 software.

### Cells sorting and single cell RNA-sequencing

Splenocytes were separately prepared from two 20-week-old female *CSF1R*-eGFP transgenic birds. *CSF1R*-eGFP^+^ cells were sorted using a BD FACS Aria IIIu sorter with the target of 5000 cells per sample. Libraries were prepared using a Chromium™ Single Cell 3’ Library & Gel Bead Kit v3 using the 10X Chromium Single Cell RNA Sequencing Platform at the University of Edinburgh. The single-cell libraries were sequenced at a depth of 50,000 reads per cells on an Illumina NovaSeq machine at Edinburgh Genomics, University of Edinburgh.

### Single cell RNA-sequencing analysis

Cell Ranger (version 3.1.0) was used to generate transcriptomic reference index from Chicken reference genome (Gallus gallus GRCg6a, Ensembl version 101) through *cellranger mkref*, and gene expression matrix through *cellranger count*. Downstream analyses were performed on R Seurat (version 3.2.2) (29). Cells with low number of features (gene; <200) and outlier counts (unique molecule identifier; <300 or >12,000), or high mitochondrial count (>40% counts from mitochondrial genes) were removed from further analysis. Principal component analysis were performed on the cells using 50 components, and the top 30 components were used for generating a network graph using Graphia (version 2.0) (30) retaining nodes and edges fulfilling the following parameters: r ≥ 0.75, knn = 10, node degree > 5, and component size > 5. The resultant cells remained in the Graphia network graph were retained in the Seurat analysis, which was subjected to further quality control using DoubletDecon to remove doublets (31).

Following quality control, cells from two samples were integrated using Seurat *SelectIntegrationFeatures*, *FindIntegrationAnchors* and *IntegrateData* functions. Cell cycle states were labelled using *CellCycleScoring* using gene list organised by Seurat based on Tirosh et al. (2016) (32). Uniform Manifold Approximation and Projection (UMAP) dimension reduction was performed across all cell types under Seurat, and Potential of Heat-diffusion for Affinity-based Transition Embedding (PHATE) (33) was used to further interrogate the DC populations. The scRNAseq data for the study is available on https://www.ncbi.nlm.nih.gov/(BioProject: PRJNA996296).

### Chicken primordial germ cell culture and transfection

PGCs were derived from embryos homozygous for the *CSF1R*-eGFP reporter transgene (28) at Hamburger–Hamilton (HH) stage 16 and expanded *in vitro* as previously described (34). Briefly, approximately 1 μl of embryonic blood was aspirated from the dorsal aorta of embryos and placed in FAot (FGF, Activin, ovotransferrin) culture medium to expand PGCs. After culturing for 2-3 weeks, PGCs were co-transfected with 2 μg of CRISPR-Cas9 vector (PX459 V2.0) (35, 36) which included two targeting guides (sgRNA) for the *XCR1* locus and a double-stranded donor plasmid (**Supplementary Table 1**) using Lipofectamine 2000 (Thermo Fisher Scientific) as described previously (37, 38). After 24 h in culture, cells were treated with 0.6 µg/ml puromycin for 48 h for selection of transfected cells. After selection, PGCs were sorted into single wells of 96-well plates using a BD FACS Aria IIIu sorter at one PGC per well. PGCs were clonally expanded for 2 to 3 weeks (36). Genomic DNA (gDNA) was extracted for genotyping as described below. Edited PGCs were cryopreserved in STEM-CELLBANKER (AMSBIO).

### Genetic screening

Genomic DNA from PGCs was extracted using QiaAmp DNA micro kit (Qiagen) according to the manufacturer’s instruction. Chorioallantoic membrane (CAM) lysate was prepared using a REDExtract-N-Amp Tissue PCR Kit (Sigma-Aldrich) and gDNA from blood was prepared using PUREGENE^®^ DNA Purification Kit (Flowgen). Primer pairs were designed to amplify the *XCR1*-transgene or wild type *XCR1* (**Supplementary Table 1**). PCR reactions included 100 ng of gDNA or CAM lysate, primer sets and REDExtract-N-Amp kit. PCR comprised the following cycling parameters: 94°C 3 min; 94°C 30 s, 58°C 30 s and 72°C 2 min for 30 cycles. Genotypes were distinguished by amplicons for the wild-type and edited allele (wild-type, monoallelic edit, and biallelic edit) (**Supplementary Figure 1**). Cultured PGCs and all birds were sexed using a W-chromosome-specific PCR (39).

### Generation of surrogate host, XCR1-iCaspase9-RFP reporter and XCR1 knockout birds

Targeted male PGC lines were thawed after storage at −150°C and cultured for 5-10 days before injection into iCaspase9 surrogate host embryos as described previously (38). One male founder was bred to wild type hens to produce birds for following studies and to generate G_1_ offspring for breeding. All G_1_ offspring were screened by PCR for the presence of iCaspase9-RFP transgene. The expression of RFP in spleen and tissues was analysed by flow cytometry or confocal microscopy. G_1_ males and females were bred to produce G_2_ offspring. The G_2_ progenies were screened by PCR for the presence of *iCaspase9-RFP* transgene or native *XCR1* gene. Homozygous (*XCR1* KO), heterozygous (HET) and wild type birds were distinguished by PCR as described above.

### Inducible ablation of XCR1^+^ cDC

B/B homodimerizer (AP20187, Takara) was injected intravenously into *XCR1*-iCaspase9-RFP reporter birds at a dose range of 0.5, 1 or 2 mg/kg, 6 birds per group. The stock solution of the drug was prepared in absolute ethanol at 62.5 mg/ml. The carrier solution was 10% PEG-400 and 2.0% Tween in sterile water. The control group received carrier solution only. Birds were sacrificed for tissues after 24 hours. Ablation rates were calculated for each dose. The optimised dose of B/B drug was then determined accordingly. For the time course, B/B homodimerizer was injected intravenously into *XCR1*-iCaspase-RFP birds at a dose of 0.5 mg/kg 6 birds per time-point. Birds were sacrificed for tissues 24 h, 48 h, 4 and 7 days after injection.

### Statistical analysis

Data were analysed using GraphPad Prism 7.00 (GraphPad, US). Statistical analysis was conducted using unpaired non-parametric Mann-Whitney test or Multiple t-test. Statistical significance was defined as follows: no significant (ns), p > 0.05, ∗, p < 0.05; ∗∗, p < 0.01; and ∗∗∗, p < 0.001.

## Results

### Dynamics and relative abundance of XCR1^+^ cDC development in the chicken spleen

**Figure 1:**
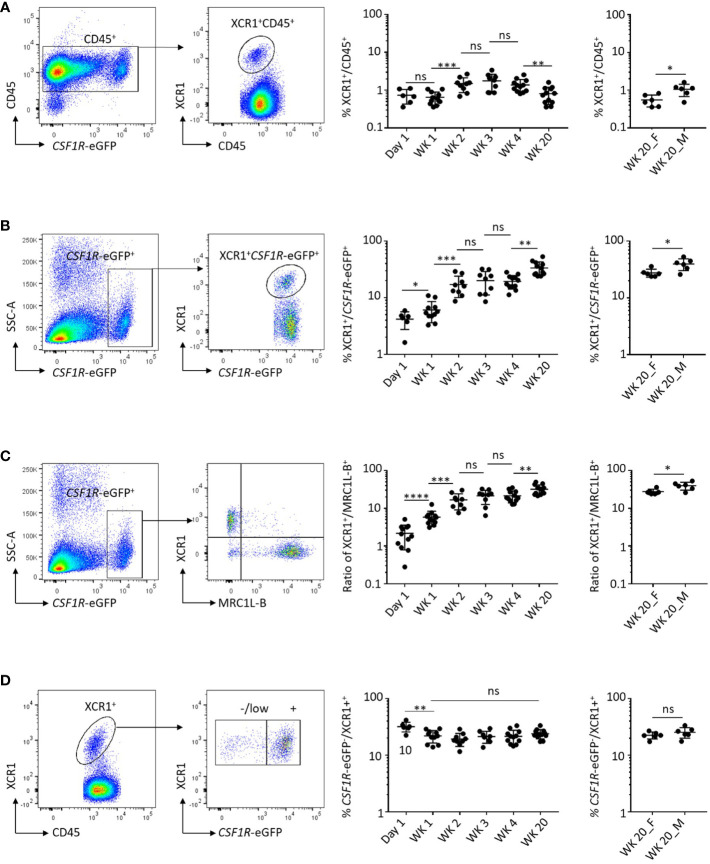
Dynamics of XCR1^+^ cDC development in the chicken spleen. **(A)** Frequency of XCR1^+^ cDCs in the chicken spleen from chicks and adult birds. Single, live and XCR1^+^ cells were gated for the analysis and expressed as a percentage of CD45^+^ cells. **(B)** Frequency of *CSF1R*-eGFP transgene expressing cells in the chicken spleen from chicks and adult birds. Single, live, CD45^+^ and *CSF1R*-eGFP transgene expressing cells were gated for the analysis and expressed as a percentage of CD45^+^ cells. Bii) Ratio of XCR1^+^ cDCs to *CSF1R*-eGFP transgene expressing cells. **(C)** Relationship of XCR1^+^ cDCs to MRC1L-B^+^ macrophages in the chicken spleen. Single, live, CD45^+^, *CSF1R*-eGFP transgene expressing XCR1^+^ or MRC1L-B^+^ cells were gated and expressed as a percentage of the *CSF1R*-eGFP transgene expressing cell population or ratios. **(D)** Percentage of the XCR1^+^ cDC population expressing the *CSF1R*-eGFP transgene. Single, live, CD45^+^, XCR1^+^ cDC were gated and expressed as a percentage of the *CSF1R*-transgene expressing cell population. Six birds per group. Chick groups were of mixed sex, whereas adult birds were separated into male and female groups. Statistical analysis was conducted using unpaired non-parametric Mann-Whitney test. Statistical significance was defined as follows: ∗p < 0·05; ∗∗p < 0·01; and ∗∗∗p < 0·001, ns, not significant.

Previously we demonstrated that the chemokine receptor XCR1 is selectively expressed on chicken cDCs and staining for XCR1 on *CSF1R*-eGFP transgene-expressing cells could be used to identify and characterise these cells (20). In this study we extend these observations by examining the dynamics of XCR1^+^ cDC development in the spleen of chickens aged between day 1 post-hatch and sexual maturity (**Figure 1**). As a percentage of the CD45^+^ cell population the XCR1^+^ cDC population was relatively stable between hatch and sexual maturity (week 20), ranging between 0.7-2.0% of CD45^+^ cells (**Figure 1A**). While the proportion of *CSF1R*-eGFP transgene-expressing cells in the CD45^+^ cell population decreased over time, likely reflecting the maturation and emergence of B and T-cell populations, the proportion of XCR1^+^ cells within the *CSF1R*-eGFP^+^ population increased from ~3% on the day of hatch to ~40% in sexually mature males (**Figures 1B, C**). Compared with sexually mature females, males consistently showed a higher percentage of XCR1^+^ splenic cDCs, expressed as either a percentage of the CD45^+^ or *CSF1R*-eGFP^+^ cell populations (**Figures 1A-C**). We found that within the splenic *CSF1R*-eGFP^+^ population the ratio of XCR1^+^ cDCs to MRC1L-B^+^ macrophages increased from ~0.025 at hatch to ~0.5 in sexually mature birds (**Figure 1C**). MRC1L-B (also known as “KUL01”) is a classical marker of chicken splenic macrophages (40, 41) and is expressed on multiple splenic macrophage subsets (20, 42). Our data therefore imply that the XCR1^+^ cDC population becomes the most abundant *CSF1R*-eGFP^+^ subset in the chicken spleen by sexual maturity. Previously (20), we showed that ~80% of XCR1^+^ cDCs expressed the *CSF1R*-eGFP transgene in three-week-old chicks. We confirmed this result and showed that the proportion of *CSF1R*-eGFP^+^ XCR1^+^ cDCs is similar in all age groups (**Figure 1D**). We also examined the relative abundance of XCR1^+^ cDCs in the ileum, Peyer’s patches (PP), skin, bone marrow (BM) and blood (**Supplementary Figures 2**, **3**). We have found that the prolonged enzymatic digestion required for extraction of cells from intestinal tissues and skin results in the loss of FLT3 staining. As such, FLT3 staining is not shown for cells derived from these tissues. XCR1^+^ cDCs are a relatively rare cell population in the BM and blood (**Supplementary Figure 2**), a minor (~1%) component of the *CSF1R*-eGFP^+^ cell population in the skin, but a major (30-40%) *CSF1R*-eGFP^+^ cell population in the ileum and PP (**Supplementary Figure 3**).

### Single-cell RNA sequencing analysis of chicken splenic dendritic cells

**Figure 2:**
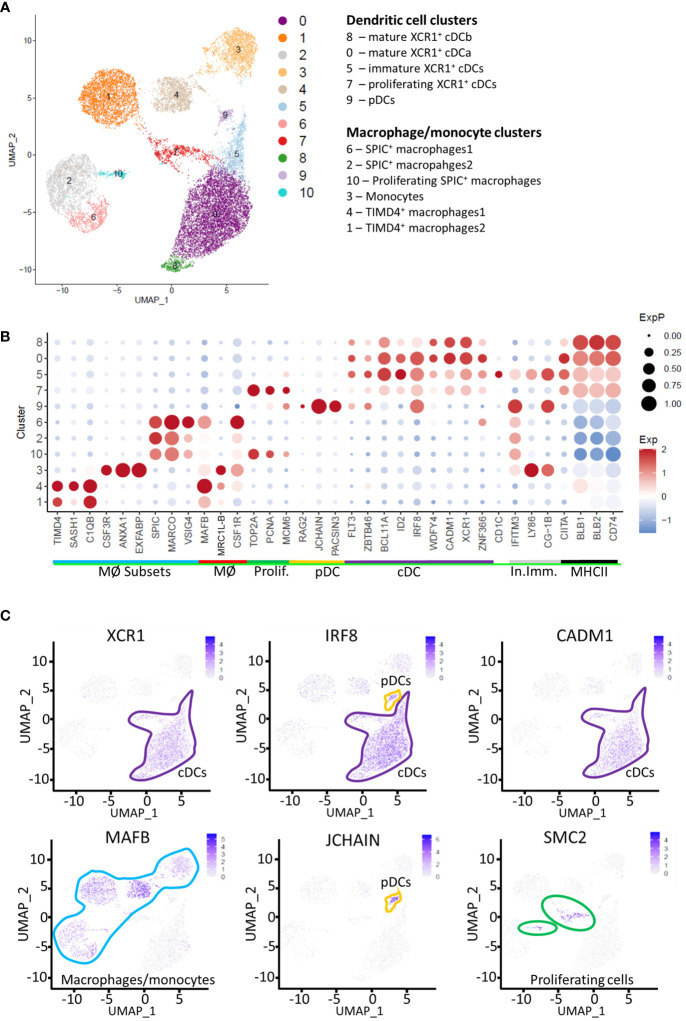
Single-cell transcriptome analysis of macrophages, monocytes and dendritic cells in the chicken spleen. **(A)** Unbiased clustering of RNA-sequence data from 16,994 *CSF1R*-eGFP transgene expressing cells derived from the spleens of two 20 week old hens. **(B)** Dotplot of selected transcript abundance in *CSF1R*-eGFP transgene expressing cell clusters. MØ Subsets, macrophage subset specific genes; MØ, pan-macrophage genes; Prolif, Proliferation genes; pDC, plasmacytoid dendritic cell genes; cDC, conventional dendritic cell genes; In. Imm, Innate immunity genes, MHCII, MHC Class II antigen presentation associated genes. **(C)** Feature plots of signature genes identifying cDCs, pDCs, macrophages/monocytes and proliferating cells.

We and others have shown that in chicken, cells that express XCR1 also express high levels of genes associated with cDCs in mice and humans (e.g. *FLT3, ZNF366, CIITA, CADM1, ID2, IRF8 and ZBTB46* (16–19);), but also the classic macrophage marker *CSF1R* (20) which is not detected or weakly detected on mammalian cDC1s (43–46). In our previous analysis of chicken splenic cDCs and macrophages (20), we identified a FLT3^HI^ cell population that expressed the dendritic cell associated marker XCR1 and lacked expression of the chicken macrophage marker MRC1L-B, suggesting a *bona fide* cDC identity. Nevertheless, unlike mammalian XCR1^+^ cDCs, this chicken cell population expressed low levels of surface CSF1R and the vast majority of this population also expressed the *CSF1R*-transgene. In addition, we also described a FLT3^LOW^ MRC1L-B^LOW^ population, which expressed high levels of CSF1R and the *CSF1R*-transgene, but lacked expression of XCR1. Both cell populations expressed high levels of surface MHCII. Previously published data suggests a macrophage/monocytic origin for the MHCII^HIGH^ MRC1L‐B^LOW^ (FLT3^LOW^) splenic cell population (42). To determine the relationship between these chicken splenic cell populations and other chicken splenic macrophage populations, which lack FLT3 expression, we performed single cell RNA-sequencing (scRNA-seq) of individual *CSF1R*-eGFP^+^ splenic cells isolated from sexually mature hens (n=2; twenty weeks of age). Across all samples, we generated 16,994 cell transcriptomes with median unique molecular identifier (UMI) and gene counts per cell of 932 and 538, respectively. For our analysis, we used two dimension-reduction techniques, Uniform Manifold and Approximation and Projection (UMAP (47);) and Potential of Heat-diffusion for Affinity-based Transition Embedding (PHATE (33);). UMAP analysis identified 10 clusters (**Figure 2**), comprising of macrophage and dendritic cells, as expected from our previous analysis of *CSF1R*-eGFP^+^ cells in the chicken spleen (**Figure 2**; **Supplementary Table 2**). Proliferating cells (predominately Clusters 7 and 10) were identified on the basis of expression of *TOP2A, PCNA, SMC2* and *MCM6* (**Figures 2B, C**) (48). Clusters were identified as macrophages on the basis of expression of genes for factors that regulate macrophage development and function (e.g. *MAFB, SPIC, CSF3R and MRC1L-B*; **Figure 2**; **Supplementary Table 2**). XCR1^+^ cDC clusters were identified on the basis of expression of *FLT3*, *XCR1, IRF8, CADM1*, and *ID2* (**Figure 2**; **Supplementary Table 2**). *MRC1L-B* (also known as *MMR1L4*) encodes the macrophage mannose receptor recognised by antibody KUL01 (41). We and others have shown that the MRC1L-B is expressed by at least two distinct macrophage populations in the chicken spleen (20, 42). In the present analysis, we found that *MRC1L-B* is expressed by three distinct cell clusters (**Figures 2B**). Collectively, *MRC1L-B* expressing clusters comprised 36.8% (6,248 of 16,994 total cells) of total cells analysed. Thus, XCR1^+^ cDCs collectively formed the largest cell subset in this analysis, comprising of 45.5% (7,734 of 16,994 total cells) of the total cells analysed. Due to the complexity of the data, functional analysis of the macrophage clusters is beyond the scope of this paper and will be detailed in a separate publication.

### Identification of chicken plasmacytoid dendritic cells

**Figure 3:**
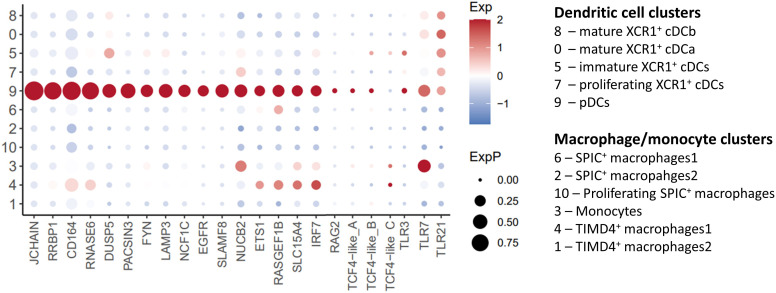
Single-cell transcriptomic identification of chicken pDCs. Dotplot of selected pDC associated genes transcript abundance in cell clusters.

**Figure 4:**
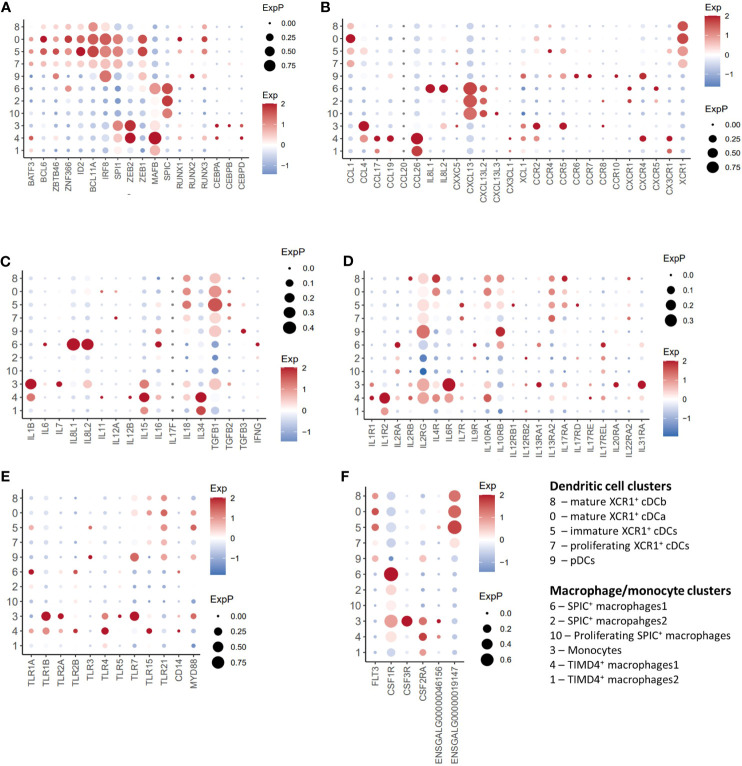
Single-cell transcriptomic analysis of transcription factor, cytokine, chemokine and TLR genes. **(A)** Dotplot of selected transcription factors transcript abundance. **(B)** Dotplot of selected chemokine/chemokine receptor transcript abundance. **(C)** Dotplot of selected cytokine transcript abundance. **(D)** Dotplot of selected cytokine receptor transcript abundance. **(E)** Dotplot of selected toll-like receptor (TLR) transcript abundance. **(F)** Dotplot of selected toll-like receptor growth factor transcript abundance.

A small cluster of *XCR1^-^ FLT3^+^ IRF8^+^* cells was noted (“Cluster 9”; **Figure 2**; **Supplementary Table 2**). On the basis of high level of expression of *JCHAIN* (49, 50) and *IRF8* (51) and the lack of expression of *ID2* and *CADM1* (51) (**Figures 2**, **3**), we putatively identified these cells as chicken plasmacytoid dendritic cells (pDCs). The transcription factors TCF4 (also known as E2-2) and ZEB2 are essential for the development of pDCs (51). We found two *TCF4* homologues in chickens ENSGALG00000055022 and ENSGALG00000033770 (referred to here as *TCF4-like_A* and *TCF4-like_B* respectively). While these genes are expressed in a subset of Cluster 9 cells (**Figure 3**), this was not at the high levels seen in mammalian pDCs. Similarly, *ZEB2* was not highly expressed in Cluster 9 cells (**Figure 4A**). In mammals, pDCs are potent producers of type I interferon (IFN) (52, 53) in response to viral pathogens via the TLR-MyD88-IRF7 pathway (54, 55). As expected, in unstimulated pDCs, we did not detect expression of type I IFN genes, but we did detect the expression of genes associated with TLR recognition of viral pathogens (*TLR3*, *TLR7* and *TLR21*) (56, 57) and type I IFN production (*IRF7*) (54), validating our identification of these cells as chicken pDCs (**Figure 3**).

### Gene expression in chicken splenic dendritic cells

Transcription factors: Chicken XCR1^+^ cDC clusters (Clusters 0, 3, 5 and 8) expressed a range of transcription factors associated with mammalian XCR1^+^ cDC development and function, including *BATF3* (58), *BLC6* (59, 60), *SPI1* (61), *ZBTB46* (62), *ZNF366* (63), *ID2* (51), *BCL11A* (64), *and IRF8* (51) (**Figures 2B**, **4A**). In contrast to mice (62), *ZBTB46* is also expressed in putative chicken pDCs (**Figures 2B**, **4A**). The macrophage associated transcription factors *SPIC, CEBPA/B/C*, and *MAFB* (46, 65) were not expressed in pDCs or XCR1^+^ cDC clusters (**Figure 2B**).

Cytokines/chemokines: As is expected in unstimulated cells, chicken pDCs and XCR1^+^ cDC clusters expressed few detectable cytokines or chemokines, with the exception of *TGFB1, IL18* and *CCL1* in XCR1^+^ cDCs, and low levels of *TGFB1* and *IL16* in pDCs (**Figures 4B, C**). With the exception of *XCR1* itself, XCR1^+^ cDC clusters did not globally express any other chemokine receptor (**Figure 4B**), although immature XCR1^+^ cDCs (Cluster 5; see below) expressed low levels of *CCR2/4/5*. pDCs expressed relatively low levels of *CCR4/5/6/7/10* and *CXCR4*. XCR1^+^ cDC clusters expressed a wide range of cytokine receptors, including *IL2RG*, *IL4R*, *IL10RA*/B, *IL13A2*, and *IL17RA* (**Figure 4D**). In contrast, cytokine gene expression in pDCs was restricted to *IL2RG* and *IL10RB* (**Figure 4D**).

TLRs: Both chicken pDCs and XCR1^+^ clusters expressed *TLR7* (albeit weakly in XCR1^+^ cDCs) and *TLR21* [a functional homologue of mammalian TLR9 (66)]. XCR1^+^ cDCs, but not pDCs expressed *TLR15* (**Figure 4E**), a TLR unique to avian, non-teleost fish, and reptilian lineages that recognises fungal-derived protease agonists (67). *TLR3* expression was restricted to pDCs (Cluster 9) and immature XCR1^+^ cDCs (Cluster 5; **Figure 4D**). Immature XCR1^+^ cDCs also expressed weak levels of *TLR1A*.

Growth factors: pDCs expressed FLT3 and the gene annotated as *CSF2RA* (ENSGALT00000026942; **Figure 4F**). *CSF1R* expression in XCR1^+^ cDC clusters was not detectable, despite the cells expressing low levels of *CSF1R* and the *CSF1R*-transgene (20). Non-proliferating XCR1^+^ cDC clusters expressed high levels of *FLT3* and the *CSF2RA* paralog ENSGALG00000019147, but not *CSF2RA* (ENSGALT00000026942).

### Substructure of the splenic XCR1^+^ cDC population

**Figure 5:**
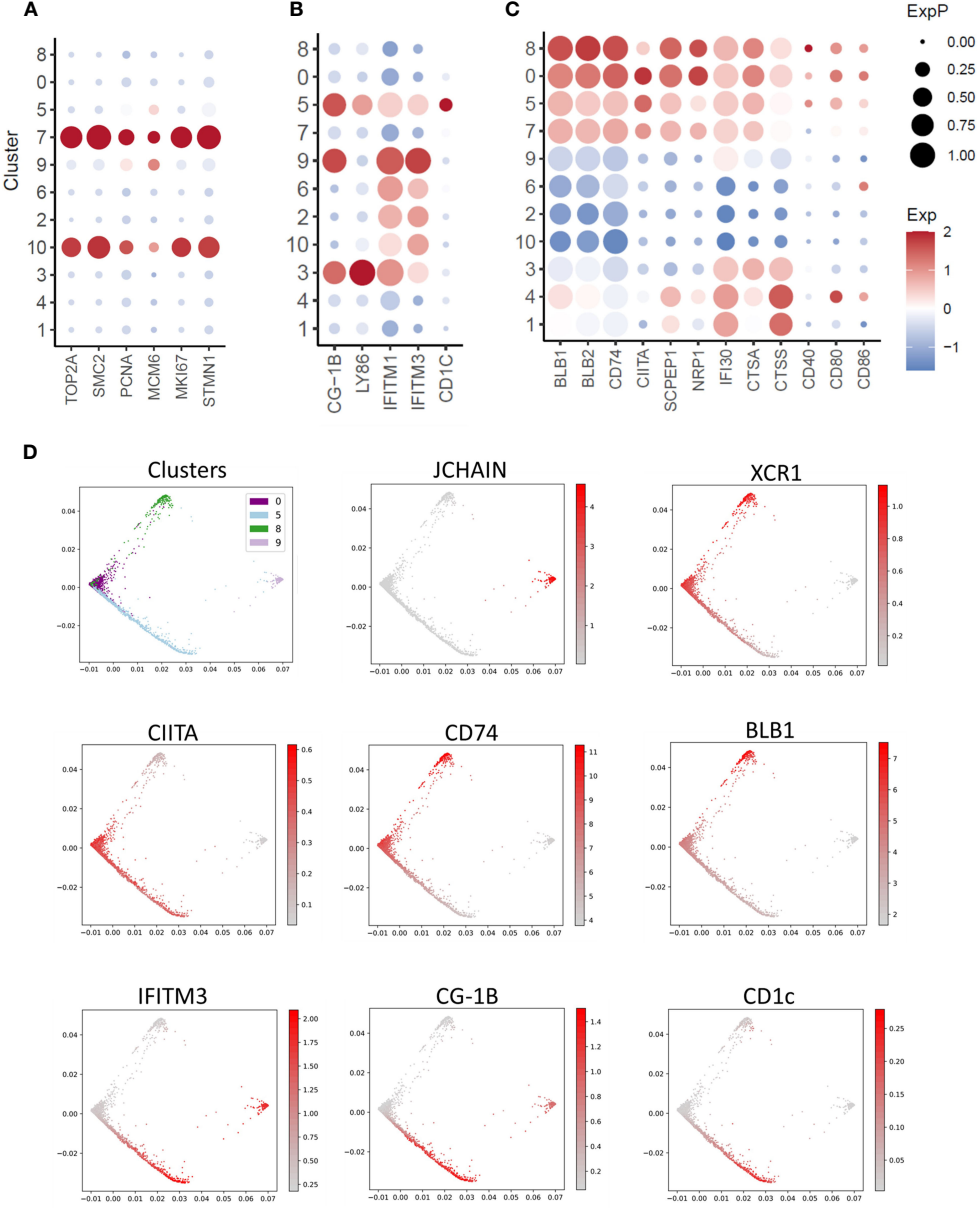
Single-cell transcriptomic analysis of cell proliferation, innate immunity and MHCII antigen presentation pathway associated genes. **(A)** Dotplot of transcript abundance for selected cell proliferation associated genes. **(B)** Dotplot of transcript abundance for selected innate immunity associated genes. **(C)** Dotplot of transcript abundance for selected MHCII antigen presentation associated genes. **(D)** PHATE map of dendritic cell clusters with feature plots of markers for pDCs (JCHAIN), cDCs (XCR1), MHCII antigen presentation (CITTA, CD74 and BLB1) and innate immunity (IFITM3, CG-1B and CD1c).

**Figure 6:**
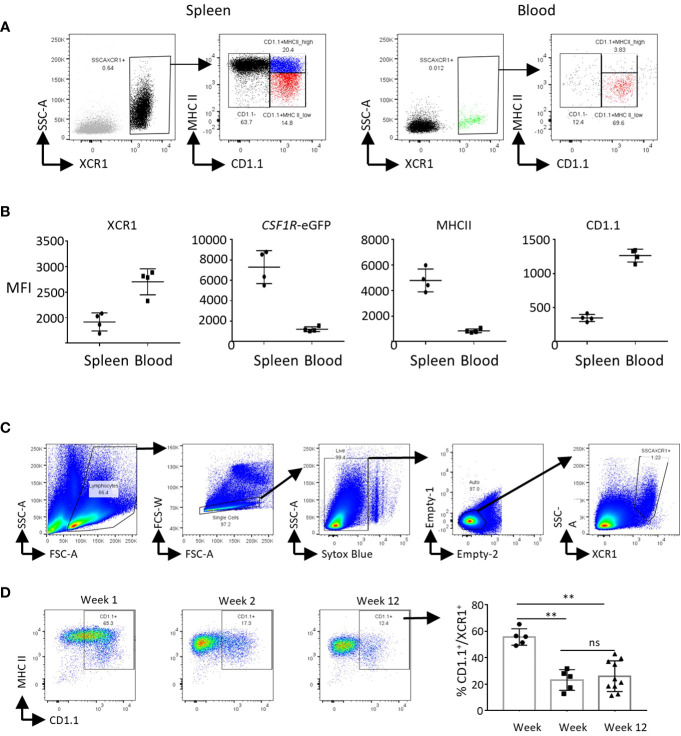
CD1.1 (CD1c) is a marker for immature splenic XCR1^+^ cDCs. **(A)** Single, live and XCR1^+^ cells from the spleen or blood were gated for flow cytometric analysis of MHCII and CD1.1 expression. **(B)** Mean fluorescence intensity of XCR1, MHCII and CD1.1 staining, and *CSF1R*-eGFP transgene expression on splenic and blood XCR1^+^ cDCs. **(C)** Flow cytometric analysis of CD1.1 and MHCII expression on splenic XCR1^+^ cDCs from chicks of difference ages.

XCR1^+^ cDC Cluster 7 expressed the cell proliferation markers *TOP2A, PCNA, MCM6, MKI67* and *STMN1* (**Figure 5A**). This represented 8.6% of XCR1^+^ cDCs (666 of 7,734 total XCR1^+^ cDCs). To confirm that chicken splenic XCR1^+^ cDCs are proliferating *in situ*, we independently determined the percentage of proliferating cells by measuring the DNA content of chicken XCR1^+^ cDCs (68). Consistent with the scRNASeq data, ~7% of XCR1^+^ cDCs were in S/G2/M phases (**Supplementary Figure 4**). Non-proliferating XCR1^+^ cDCs (Clusters 0, 5 and 8) could be differentiated from each other on basis of differential expression of genes associated with MHCII pathway antigen processing and presentation (e.g. *BLB1, BLB2, CITTA, CD40, CD80, CD86, CD74, SCPEP1, NRP1, IFI30, CTSA* and *CTSS*), or anti-viral activity (e.g. *IFITM1, IFITM3*, *LY86, CG-1B* and *CD1c*) (**Figures 5B, C**). We used PHATE to analyse the structure of the XCR1^+^ cDC population (Clusters 0, 5 and 8; **Figure 5D**), and confirm the separate developmental origin for these cells and the pDC population (Cluster 9; **Figure 5D**). Proliferating cells of different developmental origins share the expression of highly variable cell cycle genes. As this can confound trajectory analysis, we excluded proliferating XCR1^+^ cDCs (Cluster 7) from this analysis. All XCR1^+^ cDC clusters remained within the same cell trajectory, whereas pDCs were a separate population of cells. Non-proliferating XCR1^+^ cDC populations displayed a single cell trajectory with no obvious branching. The termini of the cell trajectory were represented by cells expressing high levels of genes related to MHCII antigen presentation (e.g. *CD74* and *BLB1*) or *IFITM3*, *CG-1B* and *CD1c* (**Figure 5D**). The expression of *CIITA*, the main transcription factor controlling expression MHCII genes (i.e. *BLB1* and *BLB2* encode for MHCII beta chain in the chicken), was intermediate between *BLB1* and *IFITM3* expressing cells. These data suggested a single cell population undergoing local maturation within the spleen, with immature XCR1^+^ cDCs expressing *IFITM1/3, CG-1B, LY86*, *CD1c* and relatively low levels of MHCII related genes, and mature cells expressing high levels of MHCII, but not *IFITM1/3, LY86, CG-1B* or *CD1c*. As tools to stain for IFITM1/3, LY86, CG-1B were not available at this time, to confirm the substructure within the chicken splenic XCR1^+^ cDC population we used a monoclonal antibody to chicken CD1.1 (69), which detects chicken CD1c (**Figure 6**). We found that chicken splenic XCR1^+^ cDCs could be partitioned into of CD1.1^LOW^ MHCII^HIGH^, CD1.1^HIGH^ MHCII^HIGH^ or CD1.1^HIGH^ MHCII^LOW^ subsets (**Figure 6A**), with the CD1.1^HIGH^ MHCII^LOW^ and CD1.1^LOW^ MHCII^HIGH^ representing the least and most abundant subsets respectively. In contrast, the vast majority of blood XCR1^+^ cDCs are CD1.1^HIGH^ MHCII^LOW^ (**Figures 6A, B**). As maturation of XCR1^+^ cDCs is determined by cell intrinsic and local tissue conditions (reviewed by Roquilly et al., 2022) (70), we reasoned that recently hatched chicks would contain more immature XCR1^+^ cDCs than older birds. To test this hypothesis, we determined the proportion of CD1.1^HIGH^ MHCII^LOW^ and CD1.1^LOW^ MHCII^HIGH^ XCR1^+^ cDCs in 1-week-old chicks compared to chicks aged 2 and 12 weeks (**Figure 6C**). Week-old chicks were found to have approximately 3-fold more CD1.1^HIGH^ MHCII^LOW^ XCR1^+^ cDCs than older chicks (**Figure 6D**). Taken together, these data suggest that splenic CD1.1^HIGH^ MHCII^LOW^ XCR1^+^ cDCs are immature recently migrated from the blood; and furthermore, CD1.1 (and also *IFITM1, IFITM3, LY86* and *CG-1B*) expression diminishes as MHCII expression increases in maturing splenic XCR1^+^ cDCs.

### Cross presentation

The priming of cytotoxic CD8^+^ T cells to exogenously-derived antigens is a process termed “cross-presentation”. Mammalian XCR1^+^ cDCs excel at cross-presentation of viral and tumour cell-associated antigens (9–13). As no chicken immortalised T-cell lines exist to directly test antigen cross-presentation function, we assessed the potential for antigen cross-presentation by chicken XCR1^+^ cDCs by examining the expression of genes involved in cross-presentation taken from previously published resources (**Supplementary Figure 5**). XCR1^+^ cDC clusters exhibited a range of upregulated cross-presentation genes compared to pDC or macrophage clusters. These included genes with functions directly (*WDFY4, CD74* and *PPT1*) or indirectly (*CADM1, DNASE1L3, XCR1* and *LY75*) related to cross-presentation.

### Production of the gene-edited *XCR1-iCaspase9-RFP* chicken

**Figure 7:**
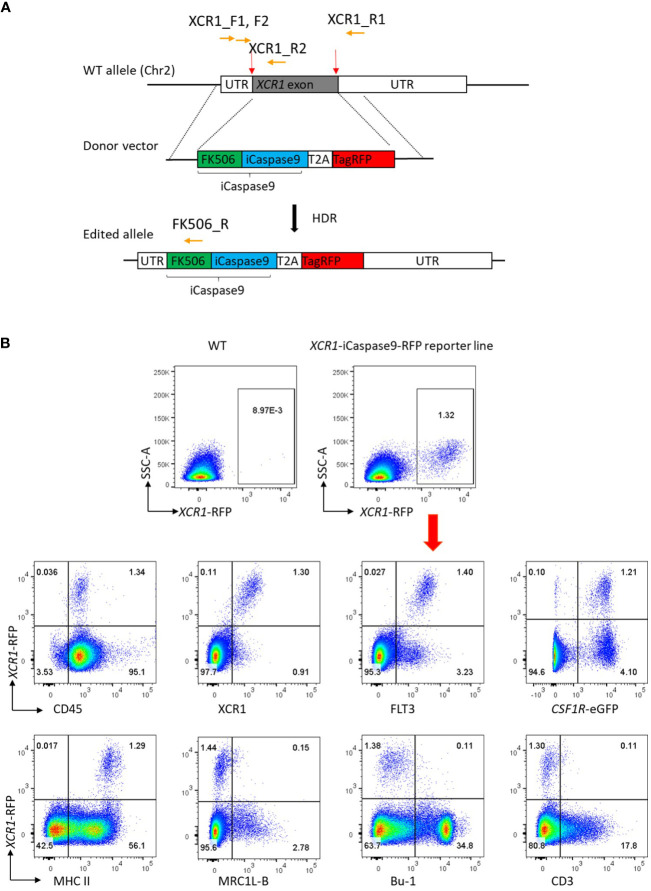
Production of the *CSF1R XCR1*-iCaspase9-RFP chicken. **(A)** CRISPR/Cas9-mediated recombination of an iCaspase9 and TagRFP (RFP) reporter gene to replace the exon at the XCR1 locus. Red arrows indicate the guide targets, orange arrows indicate the position of PCR primers used in analysis of gene targeting. **(B)** Flow cytometric analysis of splenic cells from dual transgenic *XCR1*-iCaspase9-RFP x *CSF1R*-eGFP (heterozygous for both *XCR1*-iCaspase9-RFP and *CSF1R*-eGFP transgenes). RFP expression is only detected in cells from the *XCR1*-iCaspase9-RFP chicken. RFP^+^ cells are CD45^+^, FLT3^+^, XCR1^+^, MHCIIHIGH. RFP expression is not detected in MRC1L-B^+^ (macrophage/monocyte), Bu-1^+^ (B-cells) or CD3^+^ (T-cell) populations. Representative of 20-30 animals.

Previously we developed tools to identify chicken XCR1^+^ cDCs (20). However, these did not enable specific *in situ* visualisation nor *in vivo* manipulation of XCR1^+^ cDCs. Analysis of XCR1^+^ cDCs was problematic in many tissues, as markers such as FLT3 are lost during the enzymatic digestion of tissues required for cell extraction. Therefore, we aimed to produce a gene edited chicken line in which XCR1^+^ cDCs could be visualised and conditionally ablated. Inducible caspase-9 (iCaspase9), developed as a cellular suicide gene for human stem cell therapy, is an effective system for cellular ablation in chicken embryos (38). We used CRISPR/Cas9-mediated homology-directed repair (HDR) to replace the single exon of the *XCR1* gene with an iCaspase9 construct in PGCs (**Figure 7A**). The *iCaspase9* transgene was followed by a 2A self-cleaving peptide sequence then an enhanced red fluorescent protein (*RFP*) reporter gene to mark cellular expression. Transfected *CSF1R*-eGFP transgene positive PGCs were screened and clonally expanded. 50% (20/40) of selected clones were found to have bi-allelic edits (**Supplementary Figure 1A**). A single male PGC clone with biallelic edits was selected and injected into surrogate host embryos (38). One male founder was bred to wild type hens to produce G_1_ offspring for breeding and analysis. All G_1_ offspring were screened by PCR for the presence of *iCaspase9-RFP* transgene (**Supplementary Figure 1B**). The expression of RFP expression in splenic cells of G_1_ offspring was analysed by flow cytometry (**Figure 7B**). The vast majority of RFP^+^ cells were found in the *CSF1R*-eGFP^+^ cell population, as expected for chicken XCR1^+^ cDCs (20). RFP^+^ cells were positive for the chicken cDC markers XCR1 and FLT3, expressed high levels of MHCII, but did not express markers for chicken macrophages (MRC1L-B), B-cells (Bu-1) nor T-cells (CD3), indicating the transgene was specifically expressed in chicken XCR1^+^ cDCs (**Figure 7B**). As the *XCR1*-iCaspase9-RFP transgene replaces the native XCR1 coding sequence, this has the potential to impact expression levels of XCR1. Previously we showed that XCR1 expression on chicken XCR1^+^ cDCs could be assessed by flow cytometry by measuring the binding of XCL1^AF647^ (20). We found that in birds homozygous for the *iCaspase9-RFP* transgene (i.e. deficient in XCR1) there was a total lack of binding to XCL1^AF647^; whereas heterozygous birds exhibited the same level of binding to XCL1^AF647^ as wild-type (WT) birds (**Supplementary. Figure 6A**). Heterozygous birds are therefore functionally wild-type for XCR1 expression and are suitable for assessing XCR1^+^ cDC development and function. We assessed the impact of XCR1 deficiency on splenic immune cell populations (**Supplementary Figure 6B**). Apart from a slight, but significant, decrease in B-cells (Bu-1^+^ cells) in homozygous *XCR1*-iCaspase9-RFP transgenic (i.e. XCR1 deficient; “KO”) compared to heterozygous birds (“HET”), no impact of XCR1 deficiency was noted in the CD45^+^, *CSF1R*-eGFP^+^, MRC1L-B^+^ (macrophages), CD4^+^ or CD8^+^ T-cell populations (**Supplementary Figure 6B**). As males have approximately twice as many splenic XCR1^+^ cDCs than females (**Figure 1**), we compared the number of XCR1^+^ cDCs between 20-week old *CSF1R*-eGFP *XCR1*-iCaspase9-RFP male and female chickens (**Supplementary Figure 7**). While the proportion of *CSF1R*-eGFP^+^ cells expressed as a percentage of the CD45^+^ population did not differ between the sexes, males had approximately two-fold more *XCR1*-RFP^+^ cDCs than females, expressed as either a percentage of the CD45^+^ or *CSF1R*-eGFP^+^ cell populations (**Supplementary Figure 7**).

### *XCR1*-iCaspase9-RFP transgene reporter enables detection of chicken cDCs *in situ*

**Figure 8:**
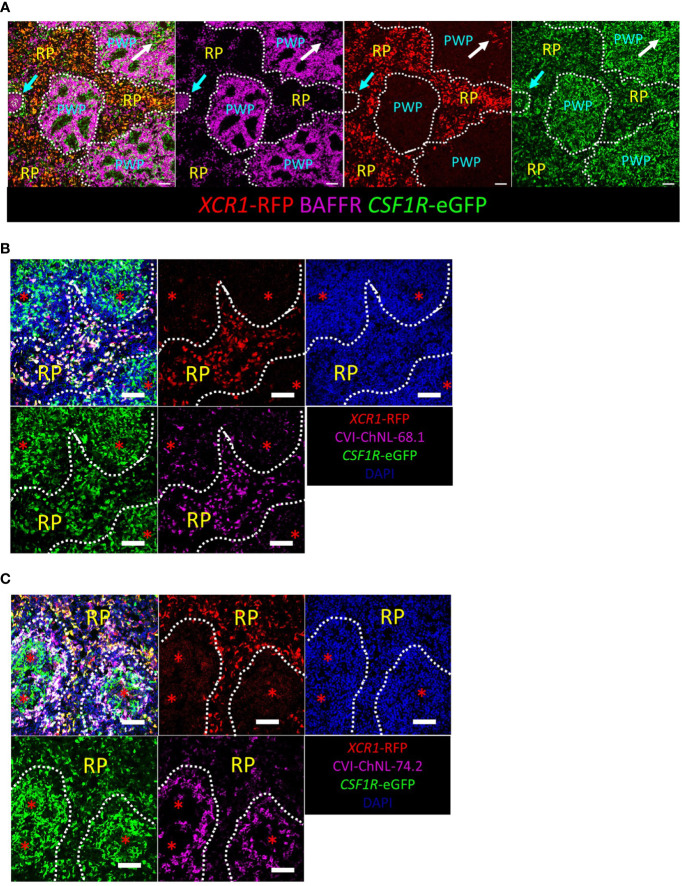
Confocal microscopic analysis of *XCR1*-RFP^+^ cDCs in the chicken spleen. Sections of spleen from dual transgenic *XCR1*-iCaspase9-RFP x *CSF1R*-eGFP were stained with anti-BAFFR **(A)** to identify the red pulp (RP) and periellipsoid white pulp (PWP). Cyan arrow = germinal centre (GC); white arrow = periarteriolar lymphoid sheaths (PALS). *XCR1*-RFP^+^ cDCs are located in the RP and PALS, but not the GC or PWP; scale bar = 50 μm. *XCR1*-RFP^+^ cells in the RP stained with the monoclonal antibody CVI-ChNL-68.1 **(B)**, but not CVI-ChNL-74.2 **(C)**. CVI-ChNL-68.1 in the PWP did not express RFP **(B)**. Red asterisk = position of the splenic ellipsoid; white dashed line = PWP/RP boundary. Scale bar = 50 μm.

RFP transgene expression was used to detect splenic XCR1^+^ cDCs *in situ* by immunofluorescence microscopy (**Figure 8**). In the spleen, RFP^+^ cells were most abundant in the red pulp and located in the periarteriolar lymphoid sheaths (PALS), rarely located in the periellipsoid white pulp (PWP), and not detected in germinal centres (GC) (**Figure 8A**). *XCR1*-RFP^+^ cDCs were widely distributed in chicken tissues (**Supplementary Figure 8**). In the small intestine (ileum and caecal tonsils, **Supplementary Figures 8A, B**), *XCR1*-RFP^+^ cDCs were abundant in the lamina propria but excluded from germinal centres (GC) and the B-cell follicles of the bursa of Fabricius (**Supplementary Figure 8F**). *XCR1*-RFP^+^ cDCs were scattered through the parenchyma of the liver and lung (**Supplementary Figures 8D, E**). In the lung, *XCR1*-RFP^+^ cDCs were found within lymphocyte clusters adjacent to the parabronchi (**Supplementary Figure 8D**). In the thymus *XCR1*-RFP^+^ cDCs were abundant in the medulla and located in the septa but excluded from the cortex (**Supplementary Figure 8C**). Previously, two monoclonal antibodies, CVI-ChNL-68.1 and CVI-ChNL-74.2, were reported to stain red pulp macrophages (71). Due to the prominent location of *XCR1*-RFP^+^ cDCs in the chicken red pulp we investigated if either of these antibodies recognised XCR1^+^ cDCs. CVI-ChNL-68.1 marked *XCR1*-RFP^+^ cDCs in the red pulp and RFP^-^ cells in PWP (**Figure 8B**). CVI-ChNL-74.2 stained a ring of RFP^-^ cells surrounding the PWP and scattered RFP^-^ cells in the red pulp (**Figure 8C**). ChNL-74.2 did not stain RFP^+^ cells.

### Chicken XCR1^+^ cDCs are closely associated with CD4^+^ T-cells in the PALS and CD8β^+^ T-cells in the splenic red pulp

**Figure 9:**
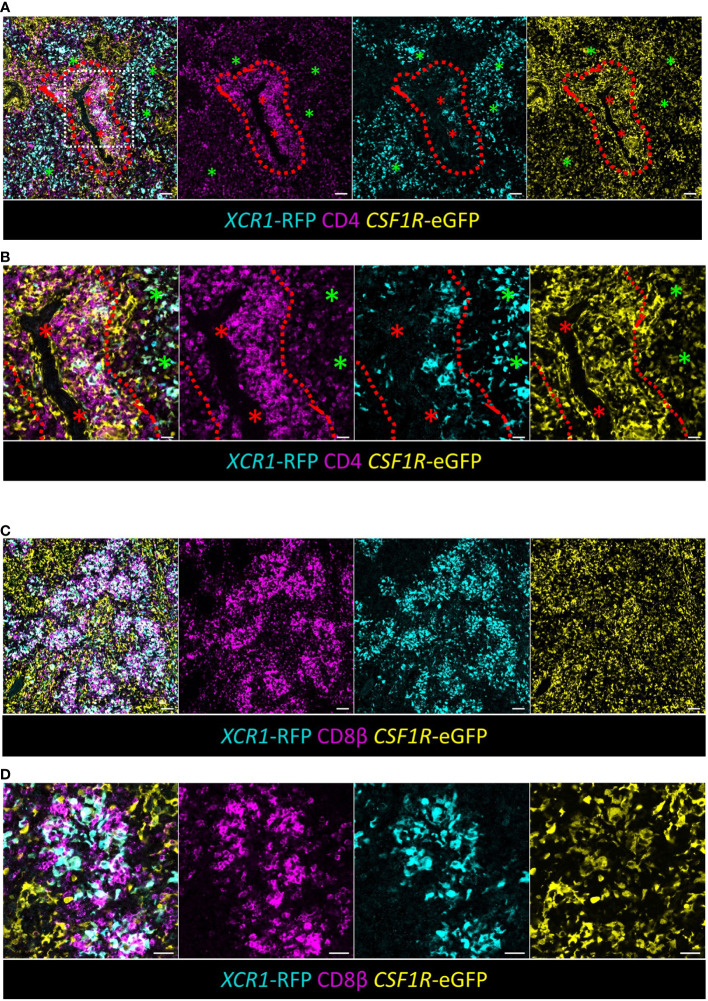
XCR1^+^ cDCs are found in close association with CD4^+^ T-cells in the PALS and CD8^+^ T-cells in the RP. **(A, B)** Sections of spleen from dual transgenic *XCR1*-iCaspase9-RFP x *CSF1R*-eGFP were stained with anti-CD4 to identify CD4^+^ T-cells. Red asterisk = artery, green asterisk = RP, dashed red line = PALS/RP boundary, scale bar = 20 μm. **(B)** A detailed area (white boxed region) in **(A)** is shown in **(B)**. Scale bar = 50 μm. **(C)** Sections of spleen from dual transgenic *XCR1*-iCaspase9-RFP x *CSF1R*-eGFP were stained with anti-CD8β to identify CD8^+^ T-cells in the RP. Scale bar = 50 μm. **(D)** A higher magnification image of the RP, showing the intimate association of *XCR1*-RFP^+^ cDCs with clusters of CD8^+^ T-cells in the RP. Scale bar = 20 μm.

Our scRNA-Seq analysis suggests that like their mammalian counter-part, chicken XCR1^+^ cDCs can present antigens to CD4^+^ T-cells via MHCII pathway and drive cytotoxic T-cell (CTL) responses by presenting exogenous antigen to CD8^+^ T-cells (known as “cross-presentation”) (72, 73). We examined the distribution of these cells by confocal microscopy (**Figure 9**). RFP^+^ cDCs were located in the RP and PALS, where they were intimately associated with CD4^+^ T^-^cells (**Figures 9A, B**). Like mice, the adult chicken spleen contains TCRαβ CD8αβ (CTLs), but chickens also have a TCRγδ CD8αβ population of cells (74, 75). CD8^+^ CTLs and TCRγδ^+^ T^-^cells can be distinguished by staining with antibodies to the chicken TCRγδ (75). Unfortunately, in our hands these antibodies did not work in the conditions required for detection of RFP. Therefore, we used a monoclonal antibody to chicken CD8β that detects both CTLs and TCRγδ^+^ CD8αβ. We found that RFP^+^cDCs were intimately associated with clusters of CD8β^+^ T-cells in the splenic RP (**Figures 9C, D**).

### Inducible ablation of chicken splenic XCR1^+^ cDCs

**Figure 10:**
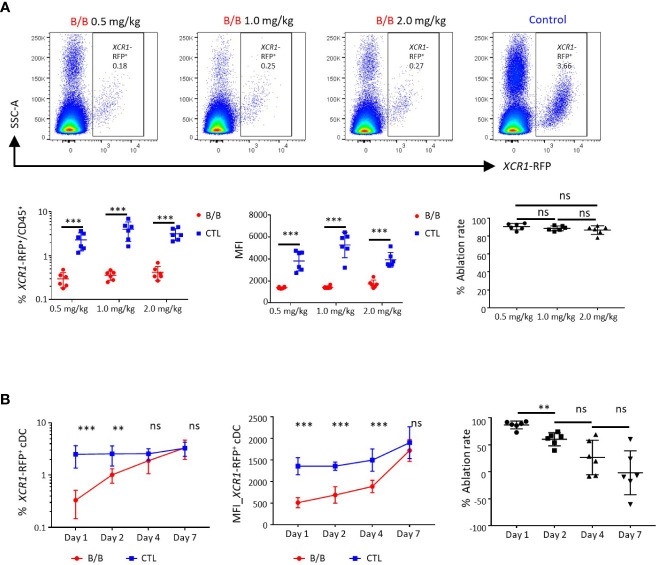
Conditional ablation of chicken *XCR1*-RFP^+^ cDCs using B/B dimerization reagent. **(A)** Confocal analysis of splenic *XCR1*-RFP^+^ cDCs from *XCR1*-iCaspase9-RFP transgenic chickens 24 hours after receiving various doses of B/B dimerization reagent or vehicle alone (Control, CTL). At all doses of B/B reagent a decrease in both the frequency of *XCR1*-RFP^+^ cDCs and the mean fluorescent intensity (MFI) of RFP expression in relation to vehicle alone was noted. **(B)** Time-course of *XCR1*-RFP^+^ cDC recovery in the spleen after a single dose of B/B dimerization reagent at a dose rate of 0.5 mg/kg. While the number of *XCR1*-RFP^+^ cDCs recovered within four days of B/B reagent administration, the MFI of RFP expression did not recover to pre-challenge levels until day 7. Six birds per group. Statistical analysis was conducted using unpaired nonparametric Mann-Whitney test for ablation rate and Multiple t-test for others. Statistical significance was defined as follows: ∗p < 0·05; ∗∗p < 0·01; and ∗∗∗p < 0·001, ns, not significant.

**Figure 11:**
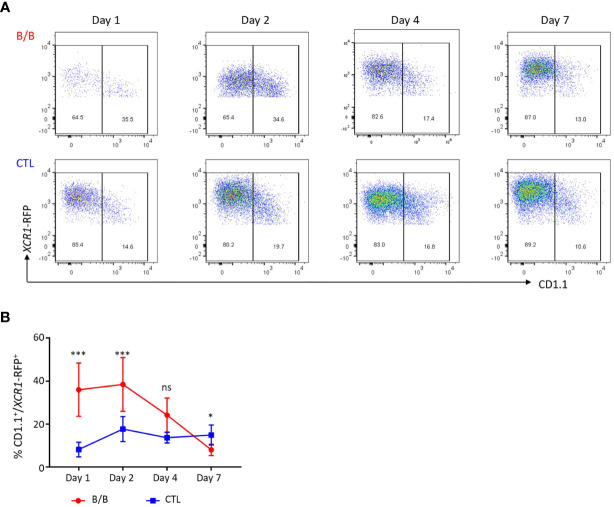
The spleen is initially repopulated by immature *XCR1*-RFP^+^ cDCs after conditional ablation. **(A, B)** Single, live and *XCR1*-RFP^+^ cells from the spleen or blood were gated for flow cytometric analysis of CD1.1 and *XCR1*-iCaspase9-RFP transgene expression. Time-course of CD1.1 and *XCR1*-iCaspase9-RFP transgene expression after a single dose of B/B dimerization reagent at a dose rate of 0.5 mg/kg. Six birds per group. Statistical analysis was conducted using Multiple t test. Statistical significance was defined as follows: ∗p < 0·05; ∗∗p < 0·01; and ∗∗∗p < 0·001, ns, not significant.

iCaspase9 has previously been used to ablate cell lineages in early chicken embryos (38). Its suitability as a system for ablating specific cell populations in post-hatch chickens was unknown. We tested the efficacy of the iCaspase9 gene under control of the chicken *XCR1* promoter for the specific ablation of chicken XCR1^+^ cDCs. Groups of six birds heterozygeous for *XCR1*-iCaspase9-RFP transgene were intravenously injected with 0.5, 1.0 or 2.0 mg/kg of the B/B dimerization drug or carrier alone (**Figure 10A**). All dosage levels were found to specifically ablate XCR1^+^ cDCs (FLT3^HIGH^ XCR1^+^ *XCR1*-RFP^+^ cells) at an ablation rate of 94-96% (**Figure 10A**; **Supplementary Figure 9A**). No effects were noted on macrophage, granulocyte, T or B-cell populations (**Supplementary Figure 9B**). Next we assessed the repopulation of the spleen by XCR1^+^ cDCs after ablation. Groups of six chickens were intravenously injected with B/B dimerization drug or carrier alone (**Figure 10B**) at a dose rate of 0.5mg/kg. Chickens were culled at 1, 2, 4 or 7 days post-injection (**Figure 10B**). After initial ablation, the spleen was rapidly repopulated by XCR1^+^ cDCs with the levels of splenic XCR1^+^ cDCs returning to the same level as in control birds by day 4-post injection. However, it was noted that the repopulating XCR1^+^ cDCs expressed lower levels of the RFP transgenic reporter, therefore despite the numbers of XCR1^+^ cDCs being normal by day 4, the mean fluorescence intensity (MFI) of the RFP expression did not return to the same level as control birds until day 7 post-injection (**Figure 10B**). We hypothesised that the reduced RFP levels observed in repopulating XCR1^+^ cDCs after ablation was due to the repopulation of spleen by immature XCR1^+^ cDCs. To test this hypothesis, we measured the levels of CD1.1 expression on XCR1^+^ cDCs after B/B reagent induced ablation (**Figure 11**). We observed that the proportion of CD1.1^HIGH^ XCR1^+^ immature cDCs increased significantly at two days post- ablation and then returned to the same levels as observed in control birds by day 4 post-ablation (**Figure 11**). In contrast, the MFI of RFP transgene expression did not return to the same levels as observed in control birds until day 7 post-ablation. (**Figure 10B**). Taken together, these data suggest that after ablation the spleen is initially repopulated by immature *XCR1*-RFP^LOW^ CD1.1^HIGH^ XCR1^+^ cDCs emigrating from the blood. Once recruited to the spleen these cells mature *in situ* to a mature CD1.1^LOW^ *XCR1*-RFP^HIGH^ phenotype, with the two markers showing different expression dynamics.

### XCR1 deficiency blocks XCR1^+^ cDC interaction with CD8^+^ T-cells in the chicken spleen

**Figure 12:**
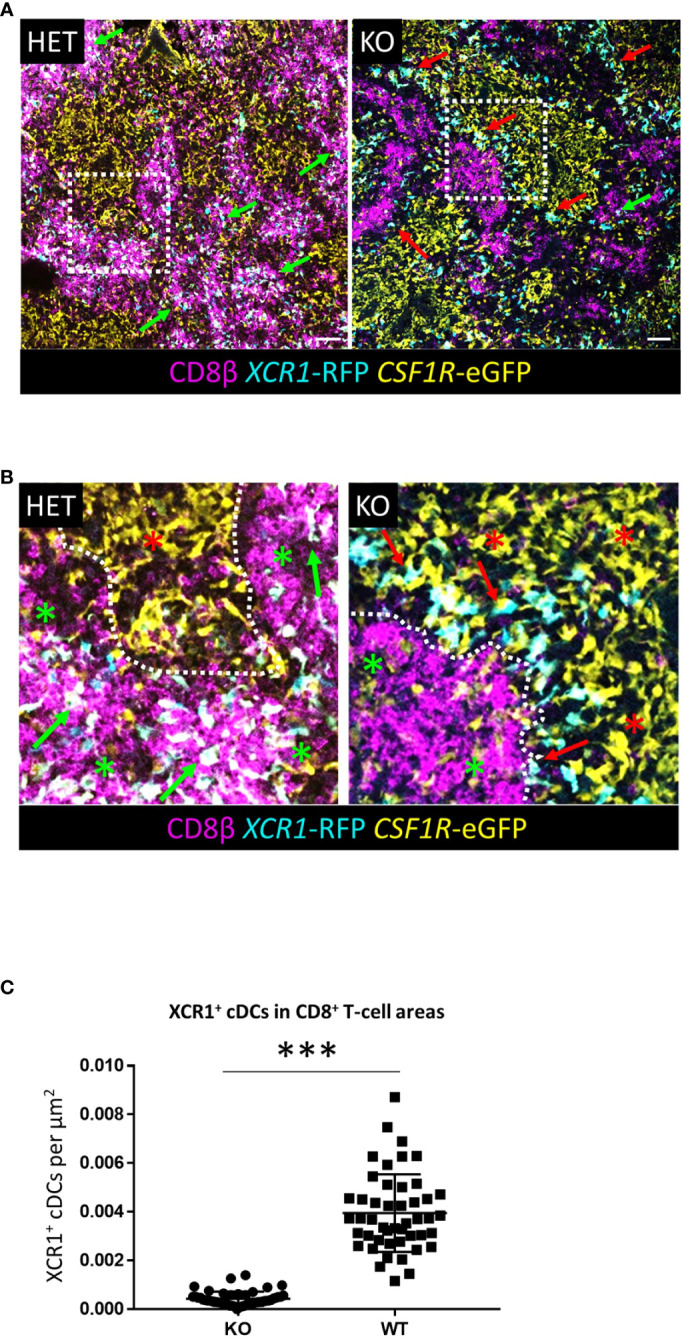
*XCR1* deficiency alters the distribution on *XCR1*-RFP^+^ cDCs in the chicken spleen. **(A, B)** Sections of spleen from *XCR1* heterozygous (HET, wild-type for *XCR1* expression) or knock-out (KO, deficient for *XCR1* expression) of dual transgenic *XCR1*-iCaspase9-RFP x *CSF1R*-eGFP were stained with anti-CD8β to identify the CD8^+^ T-cell areas in the splenic RP. **(B)** Higher magnification detail of the relative position of *XCR1*-RFP^+^ cDCs from the boxed regions in **(A)**. In HET birds *XCR1*-RFP^+^ cDCs are intimately associated with CD8^+^ T-cell clusters (green arrows) in the RP (red asterisk), whereas in KO birds the *XCR1*-RFP^+^ cDCs (red arrows) are position at the boundary (dotted line) between the red-pulp and PWP (green asterisk). **(C)** Quantification of the number of *XCR1*-RFP^+^1 cDCs found within CD8^+^ T-cell clusters. Statistical analysis was conducted using unpaired non-parametric Mann-Whitey test. Statistical significance was defined as ∗∗∗p < 0·001. 50 independent CD8^+^ T-cell clusters from three birds per group were counted.

In mice, the chemokine receptors XCR1 and CCR7 jointly control XCR1^+^ cDC migration to CD8^+^ CTLs in T-cell areas within the splenic PALS (26). Chicken XCR1^+^ cDCs did not express CCR7, indeed, with the exception of weak expression of *CCR4, CCR5* and *CX3CR1* in immature XCR1^+^ cDCs, *XCR1* itself was the only chemokine receptor expressed at high levels in these cells (**Figure 4**). The organisation of immune cell compartments of the murine spleen differs significantly from that of chickens and other vertebrates (21). Chicken CD8^+^ CTLs are predominately found in the RP (27), so we examined the impact of XCR1 deficiency on distribution of RFP^+^ cDCs in the chicken spleen. B-cells are the major immune cell population of the PWP, with a ring of CVI-ChNL-74.2^+^ macrophages demarking the boundary between the PWP and RP (**Supplementary Figures 10**, **11**). In birds heterozygous for the *XCR1*-iCaspase9-RFP transgene (wild-type (WT) for XCR1 expression) RFP^+^ cDCs were largely confined to the splenic RP (**Figure 12**, **Supplementary Figures 10**, **11**). WT RFP^+^ cDCs were rarely observed within the PWP, with the exception of the PALS. In contrast, in birds homozygous for *XCR1*-iCaspase9-RFP transgene (deficient for XCR1 expression) RFP^+^ cDCs were only occasionally located in the RP, being mostly concentrated within the PWP, intermingled with BAFFR^+^ B-cells and CVI-ChNL-74.2^+^ macrophages (**Supplementary Figures 10**, **11**). In XCR1 deficient birds, but not WT birds, RFP^+^ cDCs were also observed within the splenic ellipsoid (**Supplementary Figure 10**). We found that XCR1 deficiency results in a significant reduction in the number of RFP^+^ cDCs associated with CD8^+^ T-cell areas in the RP (**Figure 12**). Taken together, these data indicate that while XCR1 is not required for recruitment of XCR1^+^ cDCs to the chicken spleen, it is the main chemokine receptor orchestrating movement of XCR1^+^ cDCs to the CD8^+^ T-cell areas in the splenic RP.

## Discussion

Management of infectious diseases is a major challenge to poultry production in terms of economic cost, animal welfare and zoonosis that threaten human health. A plethora of avian viruses pose important challenges. Protective immune responses to viral pathogens are largely dependent on the efficient induction of antigen-specific CD8^+^ cytotoxic T lymphocytes (CTLs) and memory T-cells. Understanding the underlying immunological mechanisms that drive these T-cell responses will be required to design the next generation of poultry vaccines. XCR1^+^ cDCs are found in both mammals and chickens (16–19), and in mammalian models they drive potent CD8^+^ CTL responses (72, 73). By extension, improved vaccine responses in chickens could be achieved by manipulating cDC biology; however, basic knowledge of the biology of chicken XCR1^+^ cDCs is lacking. Here we have made significant gains in the understanding of chicken XCR1^+^ cDC biology using our previously developed chicken XCR1^+^ cDC immunological tools (20), a novel gene-edited *XCR1*-iCaspase9-RFP chicken and scRNA-seq. We provide the first detailed analysis of chicken XCR1^+^ cDC development and function. The *XCR1*-iCaspase9-RFP chicken enables both visualisation and conditional ablation of XCR1^+^ cDCs, as well as enabling the production of XCR1 knockout chickens. We confirmed our earlier observations (20) that chicken XCR1^+^ cDCs are much more abundant in comparison to their mammalian counterparts and that chickens do not have the equivalent of the mammalian cDC2 subset. XCR1 was not required for the normal development and migration of chicken cDCs to the spleen but was essential for the positioning within the spleen and clustering with CD8^+^ T-cells. Finally, we were able to differentiate between immature and mature splenic XCR1^+^ cDCs based on differential expression of genes for anti-viral activity or antigen presentation.

In mammalian studies the cDC1 (XCR1^+^) subset is the least abundant cDC subset (76). In an analysis of cDC distribution and abundance in human tissues, cDC1 frequencies are reported as 0.03-0.06% of CD45^+^ cells (77). We show here that in contrast, chicken XCR1^+^ cDCs are the most abundant population of splenic APC, with frequencies range from ~0.3-2% of total CD45^+^ cells. A similar higher abundance of XCR1^+^ cDCs was observed in the chicken small intestine, which has ~100-fold more (~3% of CD45^+^ cells) XCR1^+^ cDCs than has been reported in the human (77). We and others (17, 18, 20) have found evidence for the chicken equivalent of the mammalian XCR1^+^ cDC subset, but not the cDC2 subset. The XCR1^+^ cDC1-like and pDC-like cells have also recently been identified in teleost fish (78). This suggests that these dendritic cell subsets arose in an early vertebrate ancestor, more than 450 million years ago (79), whereas the cDC2 subset may represent a mammalian evolutionary innovation.

The existence of a single cDC subset in chickens may contribute the to the higher frequency of XCR1^+^ cDCs observed in chickens. However, the combined frequency of total human splenic cDC population remains ~20-fold less [~0.1% of CD45^+^ cells (77)] than the frequency of XCR1^+^ cDCs observed in the spleen of sexually mature chickens, indicating that factors other than the number of cDC subsets is contributing to the relative abundance of chicken XCR1^+^ cDCs. The contrasting relative abundance of cDCs between mammals and birds is likely a consequence of a fundamental difference in the composition of the secondary lymphoid organ system between mammals and all other bony vertebrates. While all bony vertebrates have spleens, complex encapsulated, tissue-draining lymph nodes are a mammalian evolutionary innovation (80). Mammalian lymph nodes act as immune hubs, ensuring efficient immune response development by increasing the likelihood of cell-cell contact between cDCs and effector cells (81–83). Our data suggests that chickens, and likely other non-mammalian vertebrates, increase the likelihood of cell-cell contact between cDCs and effector cells, by having increased numbers of cDCs within tissues that experience high levels of foreign antigen contact, such as the gut and spleen. Given birds are found on every continent and occupy diverse ecological niches where they will face similar pathogenic challenges to mammals, this alternate immune strategy is demonstrably an evolutionary success.

We noted that in addition to being more abundant than their mammalian counterparts in general, chicken XCR1^+^ cDCs are twice as abundant in the spleen of male chickens, compared to female chickens. This is potentially explained by chromosomal location of the *ZNF366*, encoding DC-SCRIPT, which controls XCR1^+^ cDC development and function (84). As chicken *ZNF366* is located on the sex-determining Z-chromosome, if expression is not completely dosage compensated, ZZ males will express more *ZNF366* than ZW females. Differences in the expression of *ZNF366* between male and female chickens have indeed been reported (85). Sex differences in resistance to infectious disease is a well-known phenomenon in chickens, which is in part due to the Z-chromosomal location of type I IFN genes in chickens (86–88). Given that type I IFN are key regulators of XCR1^+^ cDC function (89–91), it is likely that the observed sex-specific differential resistance to infectious disease is due to both qualitative and quantitative differences in XCR1^+^ cDC development and function.

Differences in local abundance of XCR1^+^ cDCs between mammals and chickens may also be due to growth factor requirements. While chicken XCR1^+^ cDCs express the developmentally critical growth factor receptor FLT3, they lack expression of CSF2R (20), which controls terminal differentiation and survival of mammalian XCR1^+^ cDCs (92). Birds also lack lymphotoxin genes (93), which are critical for the formation of lymph nodes and development of the cDC2 subset in mammals (94). We confirm here the lack of expression of the *CSF2RA* gene in chicken XCR1^+^ cDCs, and instead show high-level expression of *ENSGALG00000019147*, which encodes for a protein with homology to both mammalian CSF2RA and the interleukin-3 receptor subunit alpha (IL3RA/CD123). In humans and mice, pre-DCs express CD123, but this is lost upon differentiation into mature cDCs (95). Chicken *IL3*, encoding IL-3, is highly expressed in lymphoid organs, the small intestine and the lungs (http://animal.omics.pro/code/index.php/ChickenVar), supporting a role for IL-3 in XCR1^+^ cDC development in local tissues. Future studies to determine the role of IL-3 and other factors which support chicken XCR1^+^ cDC development and function, will be critical to the understanding of chicken XCR1^+^ cDC biology and developing methods for manipulating these cells *in vitro*.

In scRNA-seq analysis of splenic macrophages and cDCs we found that chicken splenic XCR1^+^ cDCs can be broadly divided into proliferating and non-proliferating cells. The percentage of XCR1^+^ cDCs proliferating in the chicken spleen was similar to that reported in the murine spleen (68), indicating that local proliferation in lymphoid tissues during steady state conditions is a likely conserved feature of XCR1^+^ cDCs in vertebrates. Non-proliferating XCR1^+^ cDCs could be differentiated based on opposing gradients of expression of genes related to antigen presentation and viral resistance and/or innate immune function, such as *IFITM1/3*, *CG-1B*, *LY86* and *CD1c* (96–104). We used a monoclonal antibody to chicken CD1.1 (69) (encoded by *CD1c*) in combination with anti-MHCII staining to identify this subset. Circulating XCR1^+^ cDCs uniformly showed a CD1.1^HIGH^ MHCII^LOW^ phenotype, while splenic XCR1^+^ cDCs exhibited a continuous distribution between CD1.1^HIGH^ MHCII^LOW^ to CD1.1^LOW^ MHCII^HIGH^ subsets, with the majority of cells exhibiting a CD1.1^LOW^ MHCII^HIGH^ phenotype. These data are consistent with a scenario where circulating immature CD1.1^HIGH^ MHCII^LOW^ XCR1^+^ cDCs, enter the spleen and undergo maturation, characterised by the down-regulation of viral resistance/innate immunity gene expression and up-regulating genes related to antigen presentation. In support of this, after inducible ablation of XCR1^+^ cDCs, the spleen is initially repopulated CD1.1^HIGH^ XCR1^+^ cDCs. Despite the rapid repopulation of the spleen post-ablation, mature CD1.1^LOW^ XCR1^+^ cDCs do not reach pre-ablation levels until day seven post-ablation, indicative of the maturation XCR1^+^ cDCs within the spleen. Chicks less than one week old exhibit poor responses to vaccination and possess functionally immature T- and B-cell populations (105–109). We show here that the chicken spleen contains elevated numbers of immature CD1.1^HIGH^ XCR1^+^ cDCs in the first week post-hatch. A preponderance of immature XCR1^+^ cDCs with reduced antigen presentation capacity would provide a mechanistic basis for age-dependent immune responses in chickens. As immune unresponsiveness in young chicks can be overcome by *in ovo* vaccination (110), it is likely that manipulation of XCR1^+^ cDC development and/or function in embryos/young chicks will lead to further improvements in vaccine performance.

As viral infection of cDCs may give the opportunity for some viruses to interfere with cross-presentation in cDCs (111), cDCs must balance the uptake and processing of viral antigens against the need to retain functionality. Steady-state murine cDCs employ a number of mechanisms to limit viral infection, including constitutive basal expression of antiviral IFITM1/3 (90) and high level of expression of the thioesterase, PPT1, which protects steady state DCs from viral infection by promoting antigen degradation (112). We found that immature chicken XCR1^+^ cDCs express a range of genes that potentially limit viral infection. IFITM1/3 restricts infection of chicken cells by a range of different viruses (96, 113, 114), including highly pathogenic avian influenza virus (115), and expression of chicken galectin-1 (encoded by *CG-1B*) inhibits Newcastle disease virus adsorption and replication in chicken cells *in vitro* (99). LY86 controls TLR4 signalling in mammals (116) and promotes anti-viral responses in zebrafish (101). The CD1 family of MHC related molecules participates in innate immunity/antiviral responses due to their ability to present lipid antigens to immune effectors cells, such as NK cells (102–104). Taken together, these data suggest that immature chicken XCR1^+^ cDCs may limit infection by viruses by expressing genes encoding antiviral molecules; however, optimal antigen presentation is co-incident with the down regulation in expression of these genes (112).

In mammals, pDCs are key mediators of antiviral immunity due their ability to produce large amounts of type I interferon (IFN) pathway upon detection of viral nucleic acids. As such, they are of considerable interest in the development of effective anti-viral vaccines. However, despite the identification over 60 years ago of “interferon” in influenza virus-challenged chicken embryonic chorioallantoic membranes (117), pDCs have not been formally identified in the chicken. In scRNA-seq analysis we identified chicken splenic cells expressing the dendritic associated genes *FLT3* and *IRF8*, but not the XCR1^+^ cDC associated genes *ID2, XCR1, CADM1* and *ZNF366*. As expected in unstimulated cells, we did not detect type I IFN transcripts in these cells, but we did find that these cells are enriched in expression of transcripts related to type I IFN production in pDCs. These include *TLR3, TLR7* and *TLR21*, encoding chicken TLRs which detect viral nucleic acids (57); *IRF7*, the “master regulator” of type I IFN production (118) in mammalian pDCs; as well as *FYN*, *EGFR and SLC15A4* which are all essential for TLR mediated production of type I IFN in pDCs (119–121). *PACSIN1*, which regulates the TLR7/9-mediated type I interferon responses (122), is expressed in mouse pDCs, whereas *PACSIN 1* and *3* are broadly expressed in other immune cells. In contrast, we found that *PACSIN3*, but not *PACSIN1* was highly and specifically expressed in putative chicken pDC cells, suggesting a similar role for mediating type I interferon responses in these cells. As well as genes associated with type I IFN production, we also detected other pDC associated transcripts, including *JCHAIN* (49, 50), *RAG2* (123), *RRBP1* (124), *RNASE6* (125), *SLAMF8* (126) and *RASGEF1B* (124). On this basis, we believe these cells to be *bona fide* chicken pDCs, which like their mammalian counterparts show specialisation for the recognition of viral pathogens and the production of type I IFN.

The toll-like receptor (TLR) family is a diverse and evolutionary conserved group of pattern recognition receptors (PRRs), with endosomally located TLRs 3, 7 and 9 detecting viral double-stranded RNA, single-stranded RNA and microbial DNA molecules respectively (57), whereas the other TLRs, located on the cell plasma membrane, mainly recognise microbial/fungal membrane components, such as lipids, peptidoglycans, lipoproteins, and proteins (127). In mice and humans, XCR1^+^ cDC1s express high levels of TLR3, as well as TLR7, TLR9 and TLR10 (humans only) (128, 129). However, in pigs, XCR1^+^ cDCs express TLR8 and 9, and pDC express TLR3, TLR7 and TLR9 (43). We found that chicken immature (CD1.1^HIGH^) XCR1^+^ cDCs expressed *TLR1A*, *TLR3* and *TLR21* (a functional homologue to mammalian TLR9 (66)), while mature XCR1^+^ cDCs expressed *TLR7*, *TLR15* and *TLR21*. Therefore, not only do vertebrates XCR1^+^ cDCs exhibit differences in TLR expression between species, but TLR expression differs between different XCR1^+^ cDC maturation states.

The XCR1^+^ cDC subset has an essential role in the generation of CTL responses via the cross-presentation pathway (72, 73). Cross-presentation enables presentation of exogenous antigen to CD8^+^ T cells, including cell-associated antigens from dead/apoptotic cells. Mammalian XCR1^+^ cDC1s are able to recognise and internalise apoptotic cells and/or cell fragments via the C-type lectin Clec9A/DNGR1 (130). *CLEC9A* is not present in the chicken genome, however, we previously reported that chicken XCR1^+^ cDCs express the apoptotic cell receptor TIM4, albeit at lower levels than in macrophages (17), and found that the XCR1 ligand XCL1 binds to dead cells (20). In this study, we found that chicken XCR1^+^ cDC1 also express the related apoptotic cell receptor *HAVCR1*. These data show that despite the lack of *CLEC9A*, the ability of XCR1^+^ cDCs to recognise apoptotic/dead cells is conserved in chickens. As the necessary tools to directly test the cross-presentation potential of chicken XCR1*^+^* cDCs are presently lacking, especially T-cell lines, we identified genes suggestive of a specialised role in cross-presentation in chicken XCR1^+^ cDCs. These include genes encoding for WDFY4, which is essential for cross-presentation of cell-associated antigens by cDC1 via the cytosolic pathway (131) and PPT1, which protects XCR1^+^ cDCs from viral infection by promoting antigen degradation and endosomal acidification. PPT1 expression is down-regulated after TLR stimulation to facilitate efficient cross-presentation (112). As chicken XCR1^+^ cDCs from unstimulated spleens expressed both high levels of *WDFY4* and *PPT1*, this suggests that additional signals, such as TLR signalling, may be required for efficient cross-presentation in chicken XCR1^+^ cDCs. The identification of further genes and signalling pathways related to recognition of apoptotic cells and cross-presentation in chickens XCR1^+^ cDCs will be critical to the development of novel avian vaccines to viral pathogens.

The effective generation of immune responses depends not only on the ability of cDCs to recognise specific pathogen types and/or antigens, but also on the relocation of cDCs from areas where they encounter antigens to T-cell areas of lymphoid organs where they can process and present antigen to T-cells (24, 26, 132, 133). It is known that upon activation naive CD8^+^ T cells rapidly produce XCL1 (134), resulting in clustering of XCR1^+^ cDCs and CD8^+^ T-cells, which enables T cell priming and the development of effector functions (25, 135). However, the factors that control the relocation of cDCs from areas of antigen encounter to T-cell zones in lymphoid tissues are poorly understood. In the murine spleen, XCR1^+^ cDCs are located in the RP and T-cell zone (the PALS) of the WP (25). Due to the open circulatory system of the murine spleen, cDCs and innate immune cells will initially encounter antigen/pathogens in the RP. During mouse cytomegalovirus (MCMV) infection, activated XCR1^+^ cDCs cluster in the RP with natural killer (NK) cells in a XCR1 dependent fashion (26). These activated NK cells in turn produce granulocyte-macrophage colony-stimulating factor (GM-CSF, also known as CSF2), resulting in up-regulation of CCR7 by XCR1^+^ cDCs and CCR7-dependent migration and clustering with CD8^+^ T-cells in the PALS (26). Chicken XCR1^+^ cDCs lack CSF2R expression (20) and *CCR7* expression (**Figure 4B**), suggesting that migration of XCR1^+^ cDCs to T-cell areas is controlled by different molecular signals. In addition, the organisation of lymphoid compartments in the spleen differs considerably between mice and chickens (21). In mice, the PALS is separated from the RP by surrounding B-cell follicles and a macrophage and B-cell rich marginal zone (21). In chickens, the PALS is largely composed of CD4^+^ T-cells and not separated from the RP by B-cells follicles or a marginal zone. CD8^+^ T-cells are predominately located in the RP (23). The chicken spleen has a closed circulatory system and cells, pathogens and antigens enter via fenestrated capillaries (ellipsoids) which are surrounded by a sheath of B-cells and specialised antigen trapping macrophages, the periellipsoid white pulp (PWP) (23). We show here that chicken XCR1^+^ cDCs are relatively abundant in both the RP and the PALS, where they cluster with CD8^+^ and CD4^+^ T-cells respectively, but rare in the PWP. While XCR1 deficiency does not alter splenic XCR1^+^ cDC numbers, it dramatically alters their tissue distribution in the spleen. In XCR1-deficient chickens, cDCs are found scattered within the PWP and localised to the PWP/RP boundary, but rarely observed clustering with CD8^+^ T-cells in the RP. These data suggest that XCR1 is not required for normal development or migration of chicken XCR1^+^ cDCs from the blood to the spleen, but is rather required for relocation of cDCs from the splenic PWP to RP, and clustering with CD8^+^ T-cells. These data suggest that while the expression of XCR1 on cDCs, with a specialised function of priming CD8^+^ T-cell responses through the process of antigen cross-presentation, is evolutionary conserved in vertebrates, the precise mode of action of XCL1/XCR1 differs between species. As the organisation of lymphoid compartments in the murine spleen appears to be significantly different to that of other vertebrates (including humans), it remains to be seen if the role of XCR1 in human cDC biology more closely resembles that observed in mice or in chickens.

In conclusion, we have shown that while chicken XCR1^+^ cDCs share many features in common with their mammalian counterparts, they display distinct species-specific differences. The most striking of these is the relative abundance of XCR1^+^ cDCs in chicken tissues, likely reflecting the requirements for antigen presentation in peripheral tissues and the spleen, due to the absence of lymph nodes. We also show that the *XCR1*-iCaspase9-RFP chicken is a powerful new tool for the analysis of chicken XCR1^+^ cDC development and function. We show that XCR1 is not required for normal development or migration to the spleen, but is absolutely required for the re-location of chicken cDCs to the CD8^+^ T-cell zone in the RP. Immature and mature splenic XCR1^+^ cDCs can be distinguished based on reciprocal expression of genes relating to anti-viral/innate immunity genes (e.g. *IFITM1/3*, *CG-1B*, *LY86* and *CD1c*) and antigen presentation. Finally, we show that in the first week of life post-hatch the chicken spleen is dominated by immature XCR1^+^ cDCs. This suggests that vaccination outcomes in young chicks will be improved by manipulating XCR1^+^ cDC development and function.

## Data availability statement

The data presented in this study are deposited in National Library of Medicine BioProject repository, accession number PRJNA996296. All data is publicly available: https://www.ncbi.nlm.nih.gov/bioproject/PRJNA996296.

## Ethics statement

The animal study was approved by Roslin Institute Animal Welfare and Ethical Review Board. The study was conducted in accordance with the local legislation and institutional requirements.

## Author contributions

ZW: Conceptualization, Data curation, Formal Analysis, Investigation, Methodology, Project administration, Validation, Visualization, Writing – original draft, Writing – review & editing. BS: Data curation, Formal Analysis, Investigation, Software, Validation, Visualization, Writing – review & editing. JM: Formal Analysis, Investigation, Methodology, Writing – review & editing. DM: Data curation, Formal Analysis, Investigation, Methodology, Supervision, Writing – review & editing. KH: Investigation, Methodology, Supervision, Writing – review & editing. CC-U: Investigation, Writing – review & editing. HG: Investigation, Writing – review & editing. TH: Investigation, Writing – review & editing. MB: Investigation, Writing – review & editing. NH: Investigation, Supervision, Writing – review & editing. HS: Supervision, Writing – review & editing. MS: Supervision, Writing – review & editing. MM: Conceptualization, Supervision, Writing – review & editing. AB: Conceptualization, Funding acquisition, Investigation, Methodology, Project administration, Supervision, Writing – original draft, Writing – review & editing, Formal Analysis.

## Supplementary material

Supplementary Table 1 Sequences for single guide RNA (sgRNA), donor DNA and primers.

Supplementary Table 2 Summary of the identified clusters from Single-cell RNA sequencing analysis of chicken splenic dendritic cells.

Supplementary Figure 1 PCR screening of edited PGC and gene edited chickens.

Supplementary Figure 2 Flow cytometric analysis of chicken XCR1^+^ cDCs in the blood and bone-marrow (BM).

Supplementary Figure 3 Flow cytometric analysis of chicken XCR1^+^ cDCs in the spleen, ileum, Peyer’s Patch and skin.

Supplementary Figure 4 Quantification of proliferation of XCR1^+^ cDCs in the chicken spleen.

Supplementary Figure 5 Expression of cross-presentation related genes.

Supplementary Figure 6 Analysis of the impact of *XCR1* gene knockout on XCR1 expression and relative numbers of *XCR1*-RFP^+^ cDCs and various selected splenic cell populations.

Supplementary Figure 7 Male birds contain a higher proportion of splenic XCR1^+^ cDCs than female birds.

Supplementary Figure 8 Confocal analysis of *XCR1*-RFP^+^ cDCs in chicken tissues.

Supplementary Figure 9 Impact of B/B dimerization reagent ablation of *XCR1*-RFP^+^ cDC on selected splenic cell populations.

Supplementary Figure 10 Impact of XCR1 deficiency on the distribution of *XCR1*-RFP^+^ cDCs in the chicken spleen; B-cell staining.

Supplementary Figure 11 Impact of XCR1 deficiency on the distribution of *XCR1*-RFP^+^ cDCs in the chicken spleen; Macrophage staining.
