# Supplementary material for: Development and function of chicken XCR1+ conventional dendritic cells
Source: Front Immunol. 2023 Oct 25;14:1273661. doi: 10.3389/fimmu.2023.1273661 (PMC10634274; doi:10.3389/fimmu.2023.1273661)
Supplement: Supplementary Table 1 — Sequences for single guide RNA (sgRNA), donor DNA and primers. [file Table_1.docx]

Supplementary Table 1. Sequences for single guide RNA (sgRNA), donor DNA and primers

| Item | sequence |
| --- | --- |
| sgRNA guide_1 | Sense: CACCGTAATTGTCATCCCAACCAGA  Anti-sense: AACTCTGGTTGGGATGACAATTAC |
| sgRNA guide_2 | Sense: CACCGAGATGGACGCATCTTCATAG  Anti-sense: AAACCTATGAAGATGCGTCCATCTC |
| Donor DNA sequence | ATGGGCGTGCAGGTGGAGACTATCTCCCCAGGAGACGGGCGCACCTTCCCCAAGCGCGGCCAGACCTGCGTGGTGCACTACACCGGGATGCTTGAAGATGGAAAGAAAGTTGATTCCTCCCGGGACAGAAACAAGCCCTTTAAGTTTATGCTAGGCAAGCAGGAGGTGATCCGAGGCTGGGAAGAAGGGGTTGCCCAGATGAGTGTGGGTCAGAGAGCCAAACTGACTATATCTCCAGATTATGCCTATGGTGCCACTGGGCACCCAGGCATCATCCCACCACATGCCACTCTCGTCTTCGATGTGGAGCTTCTAAAACTGGAATCTGGCGGTGGTTCCGGAGTCGACGGATTTGGTGATGTCGGTGCTCTTGAGAGTTTGAGGGGAAATGCAGATTTGGCTTACATCCTGAGCATGGAGCCCTGTGGCCACTGCCTCATTATCAACAATGTGAACTTCTGCCGTGAGTCCGGGCTCCGCACCCGCACTGGCTCCAACATCGACTGTGAGAAGTTGCGGCGTCGCTTCTCCTCGCTGCATTTCATGGTGGAGGTGAAGGGCGACCTGACTGCCAAGAAAATGGTGCTGGCTTTGCTGGAGCTGGCGCGGCAGGACCACGGTGCTCTGGACTGCTGCGTGGTGGTCATTCTCTCTCACGGCTGTCAGGCCAGCCACCTGCAGTTCCCAGGGGCTGTCTACGGCACAGATGGATGCCCTGTGTCGGTCGAGAAGATTGTGAACATCTTCAATGGGACCAGCTGCCCCAGCCTGGGAGGGAAGCCCAAGCTCTTTTTCATCCAGGCCTGTGGTGGGGAGCAGAAAGACCATGGGTTTGAGGTGGCCTCCACTTCCCCTGAAGACGAGTCCCCTGGCAGTAACCCCGAGCCAGATGCCACCCCGTTCCAGGAAGGTTTGAGGACCTTCGACCAGCTGGACGCCATATCTAGTTTGCCCACACCCAGTGACATCTTTGTGTCCTACTCTACTTTCCCAGGTTTTGTTTCCTGGAGGGACCCCAAGAGTGGCTCCTGGTACGTTGAGACCCTGGACGACATCTTTGAGCAGTGGGCTCACTCTGAAGACCTGCAGTCCCTCCTGCTTAGGGTCGCTAATGCTGTTTCGGTGAAAGGGATTTATAAACAGATGCCTGGTTGCTTTAATTTCCTCCGGAAAAAACTTTTCTTTAAAACATCAGTCGACGGATCCGGAGAGGGCAGAGGAAGTCTGCTAACATGCGGTGACGTCGAGGAGAATCCTGGACCTATGGTGTCTAAGGGCGAAGAGCTGATTAAGGAGAACATGCACATGAAGCTGTACATGGAGGGCACCGTGAACAACCACCACTTCAAGTGCACATCCGAGGGCGAAGGCAAGCCCTACGAGGGCACCCAGACCATGAGAATCAAGGTGGTCGAGGGCGGCCCTCTCCCCTTCGCCTTCGACATCCTGGCTACCAGCTTCATGTACGGCAGCAGAACCTTCATCAACCACACCCAGGGCATCCCCGACTTCTTTAAGCAGTCCTTCCCTGAGGGCTTCACATGGGAGAGAGTCACCACATACGAAGACGGGGGCGTGCTGACCGCTACCCAGGACACCAGCCTCCAGGACGGCTGCCTCATCTACAACGTCAAGATCAGAGGGGTGAACTTCCCATCCAACGGCCCTGTGATGCAGAAGAAAACACTCGGCTGGGAGGCCAACACCGAGATGCTGTACCCCGCTGACGGCGGCCTGGAAGGCAGAAGCGACATGGCCCTGAAGCTCGTGGGCGGGGGCCACCTGATCTGCAACTTCAAGACCACATACAGATCCAAGAAACCCGCTAAGAACCTCAAGATGCCCGGCGTCTACTATGTGGACCACAGACTGGAAAGAATCAAGGAGGCCGACAAAGAGACCTACGTCGAGCAGCACGAGGTGGCTGTGGCCAGATACTGCGACCTCCCTAGCAAACTGGGGCACAAACTTAATTGA |
| Primers to amply *XCR1* region | XCR1_F1: CTTGGCACCCGAGGTACTTA; XCR1_R1: TCAGGCAAGAAAGAAATGTGC; Product size: 2664 (transgene); 1666 (wt) |
| Primers to amply *XCR1* region | XCR1_F2: CTTGGAGGTAGCTGCTGGAGTTT; XCR1_R1: TCAGGCAAGAAAGAAATGTGC; Product size: 2375 (transgene); 1377 (wt) |
| Primers to amply native *XCR1* gene only | XCR1_F1:CTTGGCACCCGAGGTACTTA; XCR1_R2: CAGCATGCACGAGAAGACTA; Product size: 590bp |
| Primers to amply *XCR1*-icaspase9-RFP transgene only | XCR1_F1:CTTGGCACCCGAGGTACTTA; FK506_R: TTCAAGCATCCCGGTGTAGT; Product size: 419bp |
