# Supplementary material for: Development and function of chicken XCR1+ conventional dendritic cells
Source: Front Immunol. 2023 Oct 25;14:1273661. doi: 10.3389/fimmu.2023.1273661 (PMC10634274; doi:10.3389/fimmu.2023.1273661)
Supplement: Supplementary Table 2 — Summary of the identified clusters from Single-cell RNA sequencing analysis of chicken splenic dendritic cells. [file Table_2.docx]

Supplementary Table 2: Summary of the identified clusters from Single-cell RNA sequencing analysis of chicken splenic dendritic cells

| **Cluster** | **Cell type** | **Signature genes** | **References** |
| --- | --- | --- | --- |
| 0 | Mature XCR1^+^ cDCs1 | *FLT3, ID2, IRF8, CADM1, XCR1, BLB1/2*^HIGH^ | 16-19 |
| 1 | TIMD4^+^ macrophages2 | *TIMD4, MAFB, MRC1L-B* | 17, 40-42, 46 |
| 2 | SPIC^+^ macrophages1 | SPIC, *MAFB, MRC1L-B, CSF1R* | 20, 40-42, 46, 65 |
| 3 | Monocytes | CSF1R, CSF3R, MAFB, MRC1L-B | 20, |
| 4 | TIMD4^+^ macrophages1 | *TIMD4, MAFB, MRC1L-B, CSF1R* | 17, 20, 40-42, 46 |
| 5 | Immature XCR1^+^ cDCS | *FLT3, ID2, IRF8, CADM1, XCR1, BLB1/2^LOW^* | 16-19 |
| 6 | SPIC^+^ macrophages2 | SPIC, *MAFB, MRC1L-B, CSF1R* | 20, 40-42, 46, 65 |
| 7 | Proliferating XCR1^+^ cDCs | *FLT3, ID2, IRF8, CADM1, XCR1, TOP2A, PCNA, MCM6* | 16-19, 48 |
| 8 | Mature XCR1^+^ cDCS2 | *FLT3, ID2, IRF8, CADM1, XCR1, BLB1/2*^HIGH^ | 16-19 |
| 9 | pDCs | *FLT3, JCHAIN, IRF8* | 48-50 |
| 10 | Proliferating SPIC^+^ macrophages | SPIC, CSF1R, *TOP2A, PCNA, MCM6* | 20, 40-42, 46, 48 |
