# Supplementary material for: Development and function of chicken XCR1+ conventional dendritic cells
Source: Front Immunol. 2023 Oct 25;14:1273661. doi: 10.3389/fimmu.2023.1273661 (PMC10634274; doi:10.3389/fimmu.2023.1273661)

Supplementary Figure 1:

A

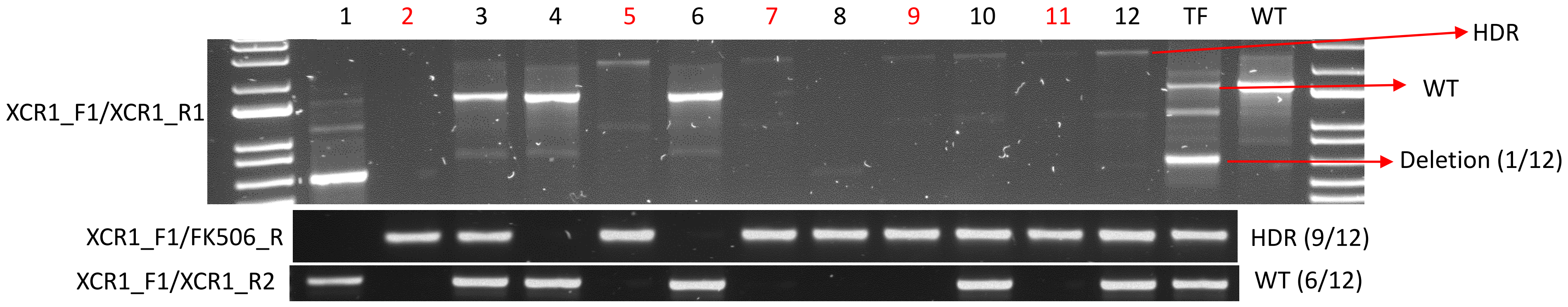

B

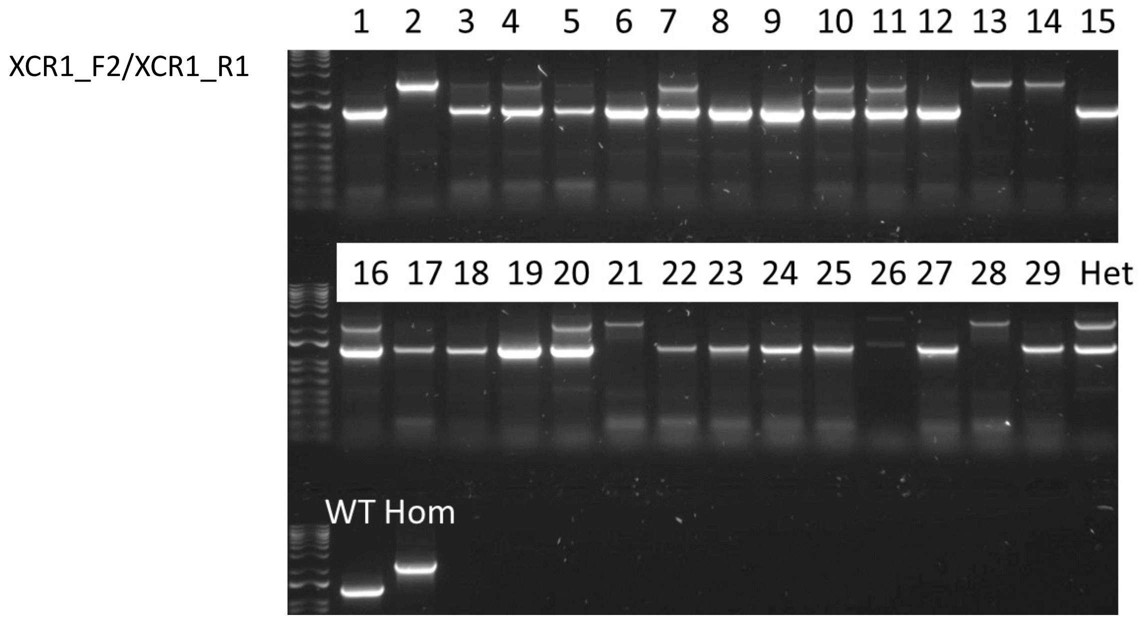

Supplementary Figure 2

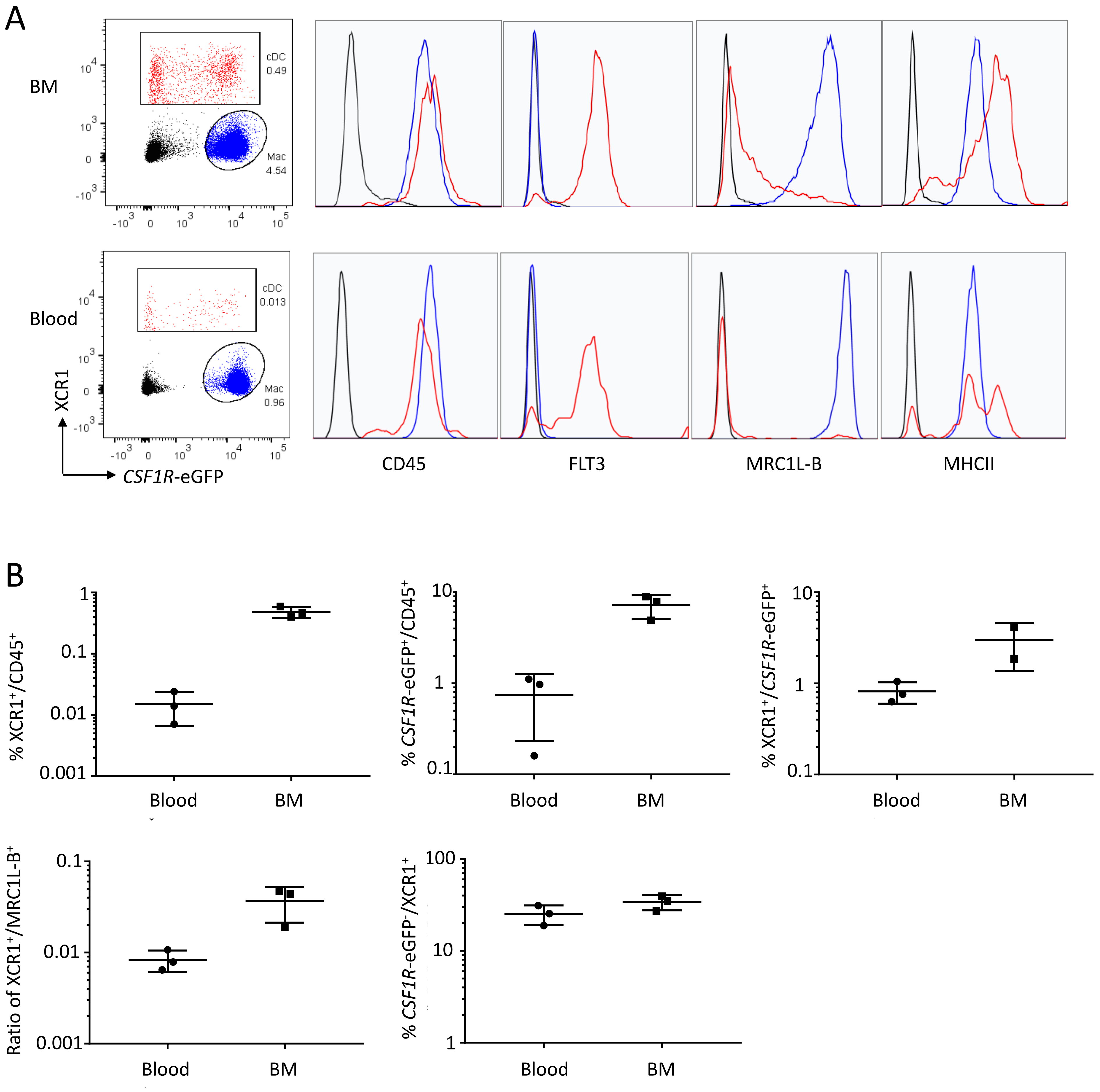

Supplementary Figure 3

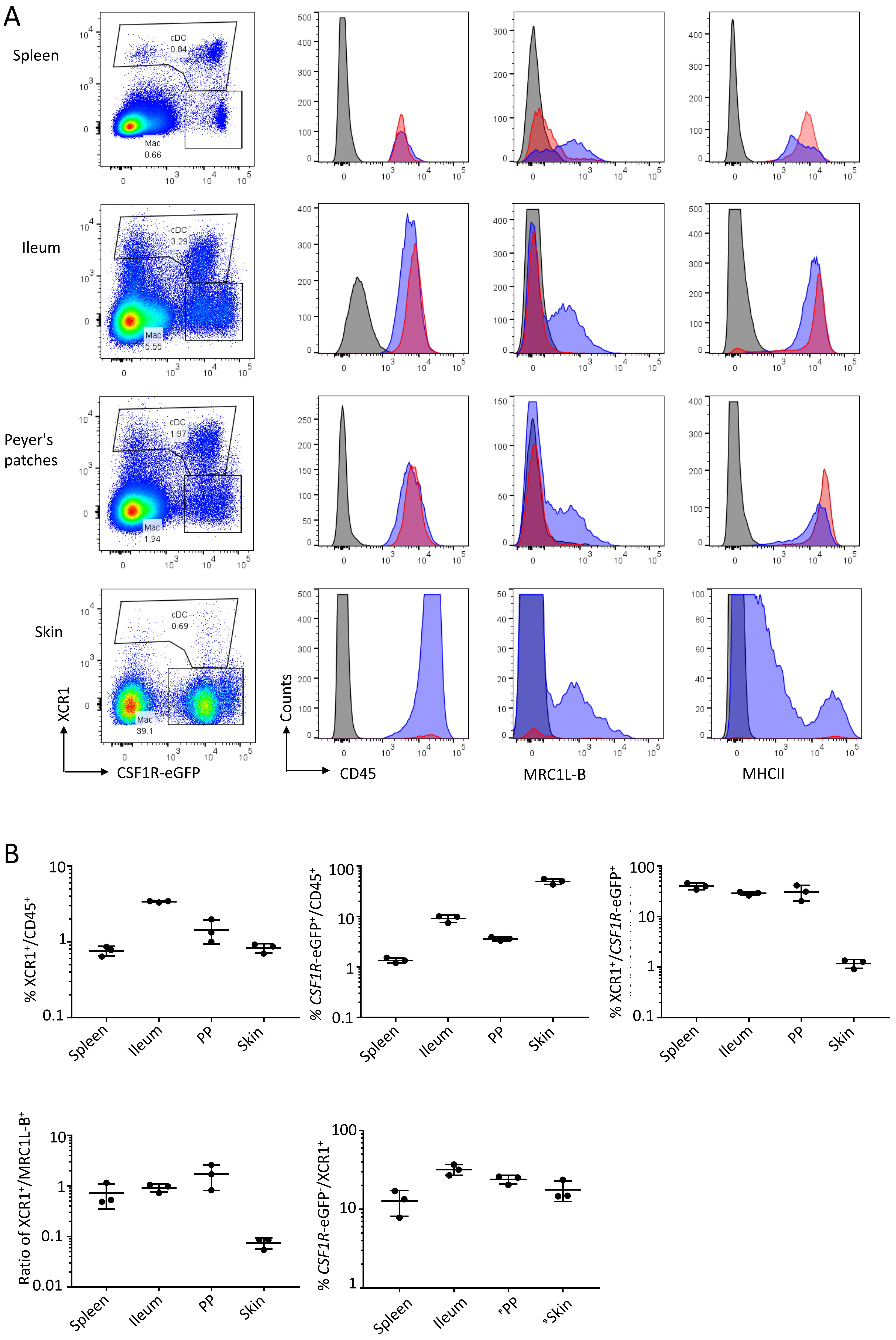

Supplementary Figure 4

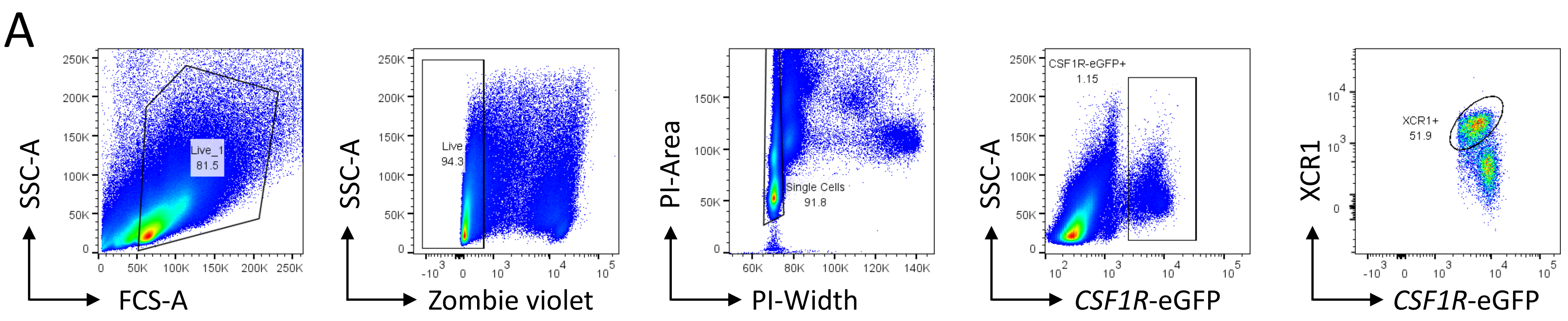

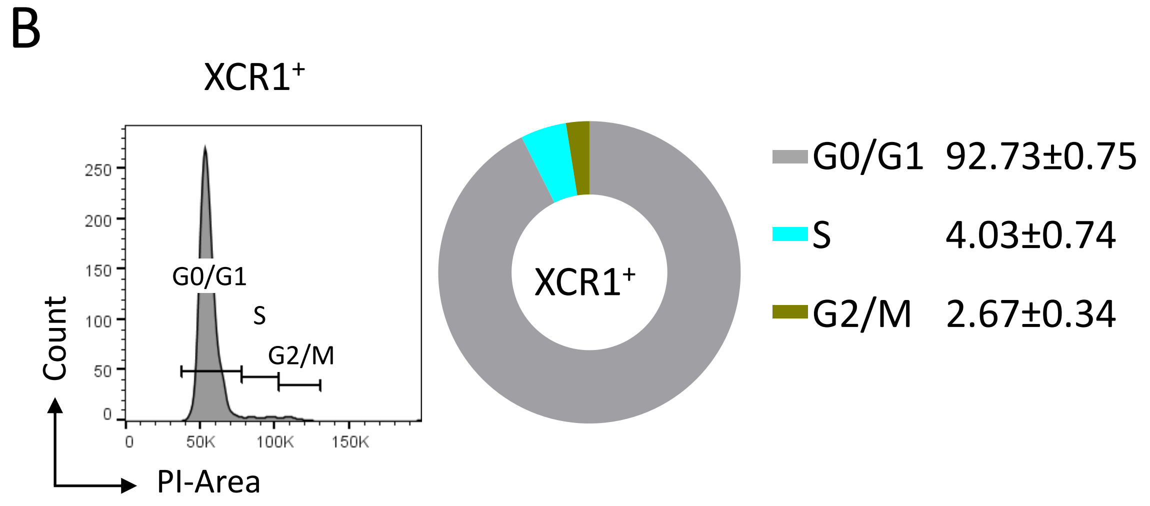

Supplementary Figure 5

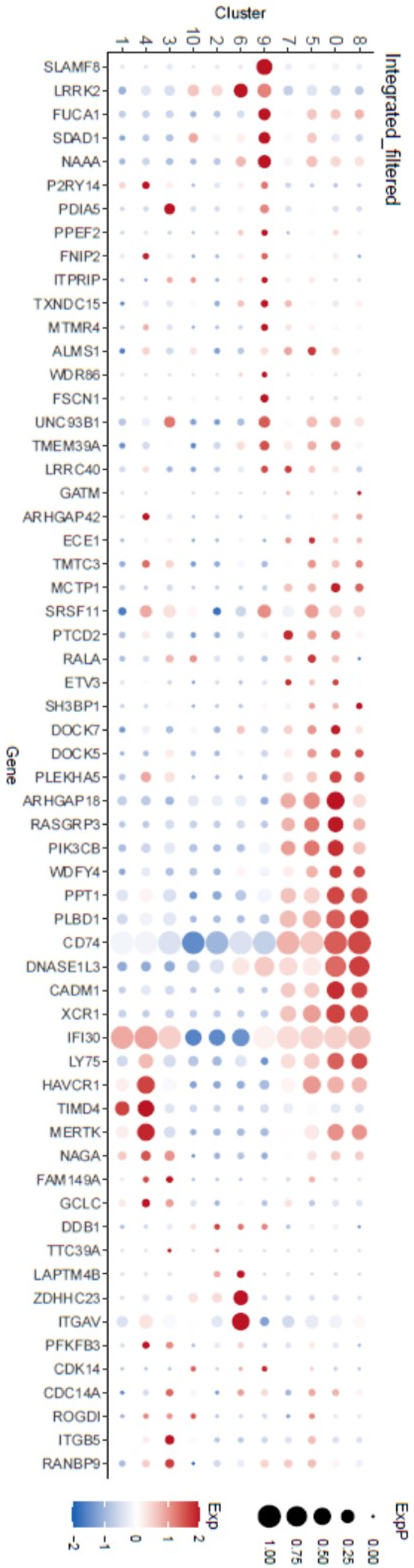

Supplementary Figure 6

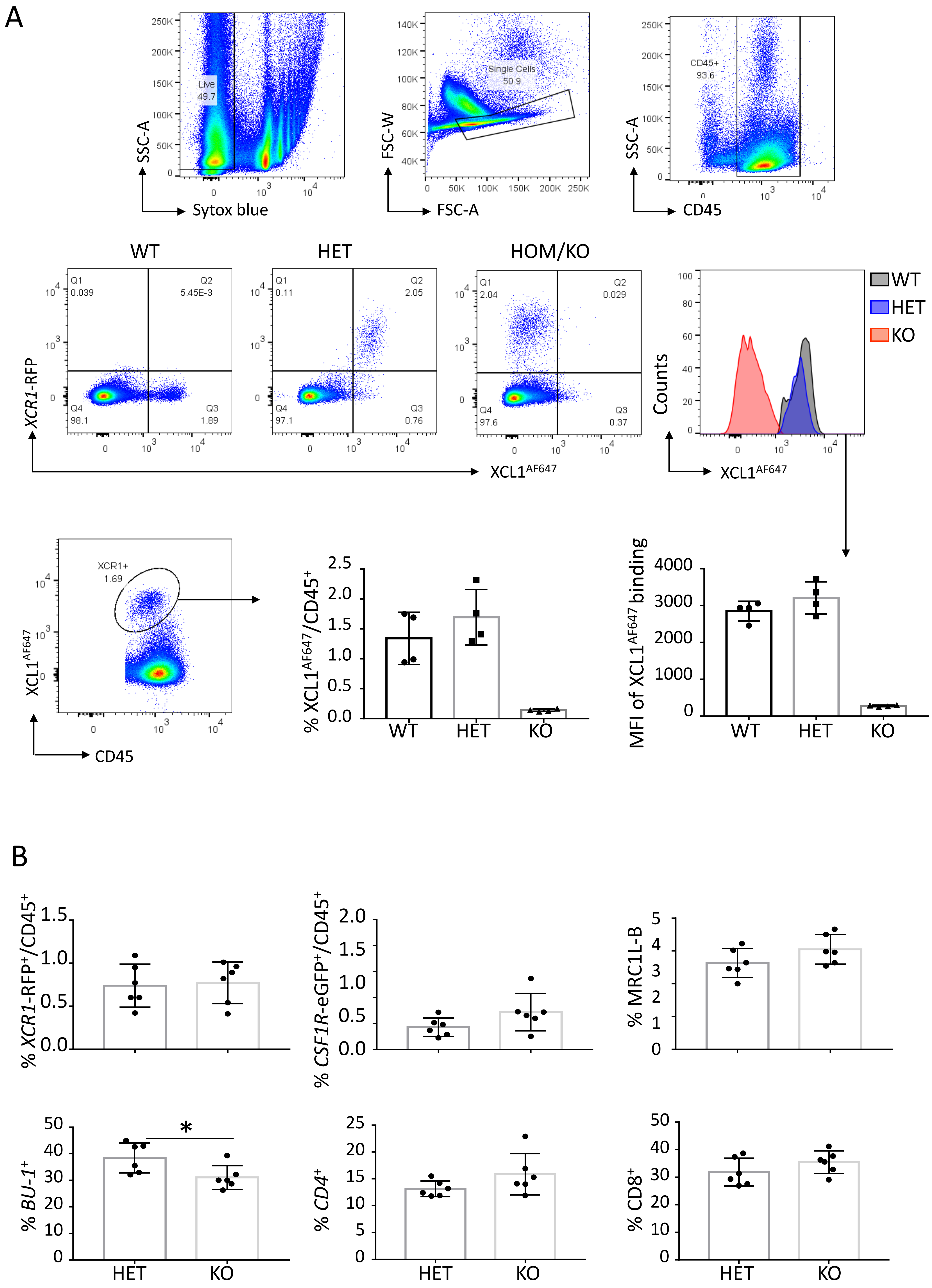

Supplementary Figure 7

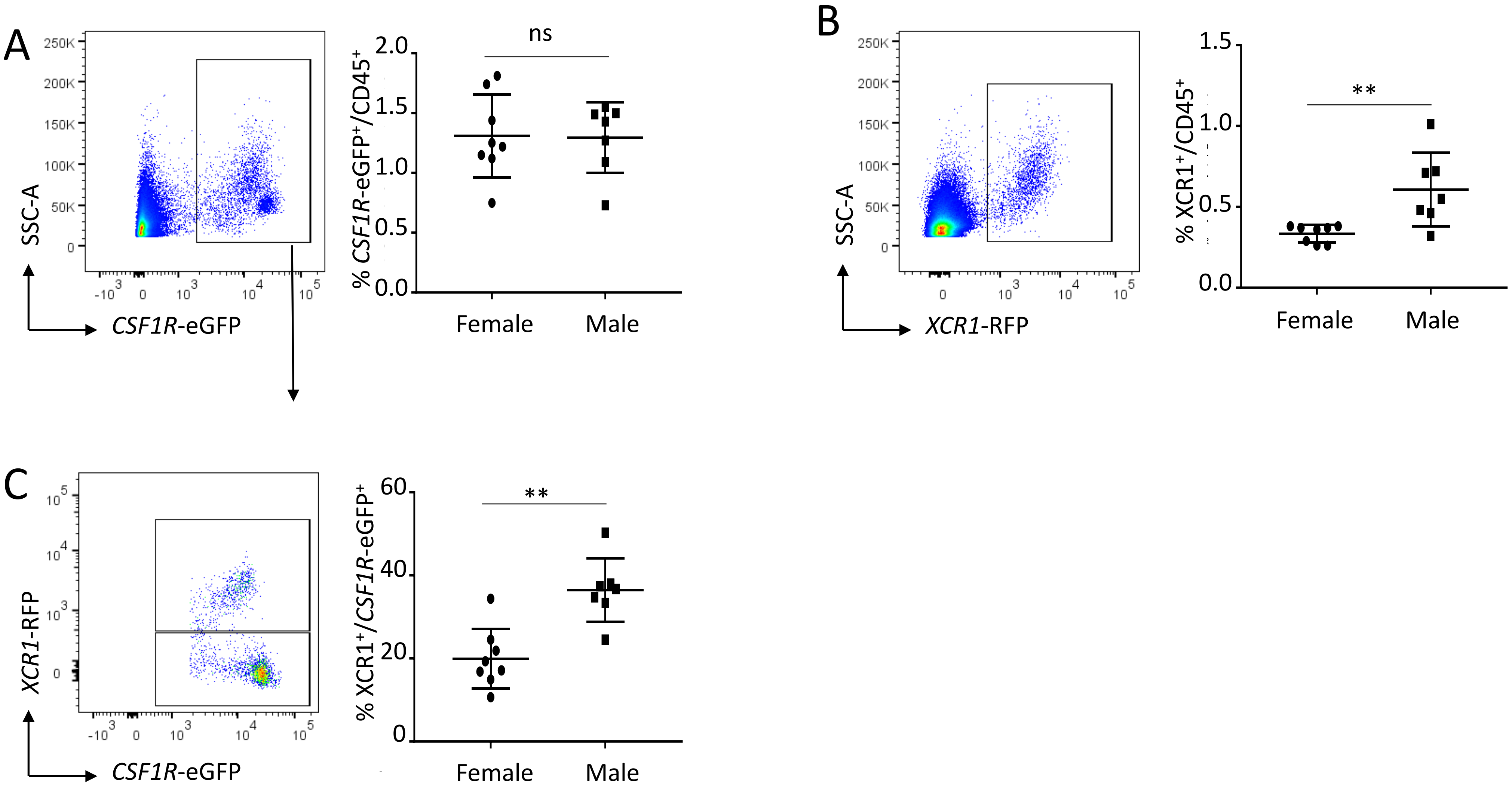

Supplementary Figure 8

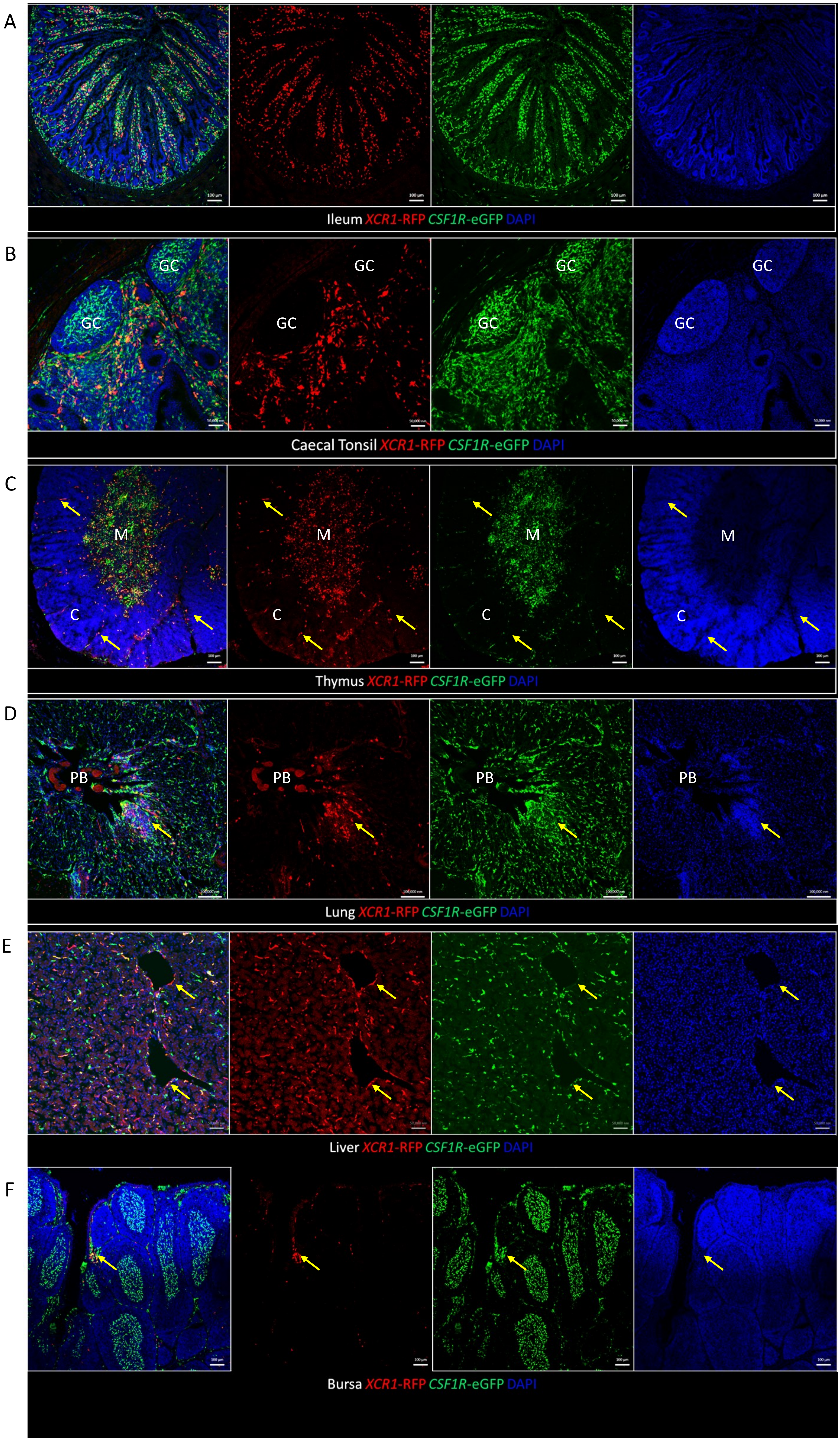

Supplementary Figure 9

A

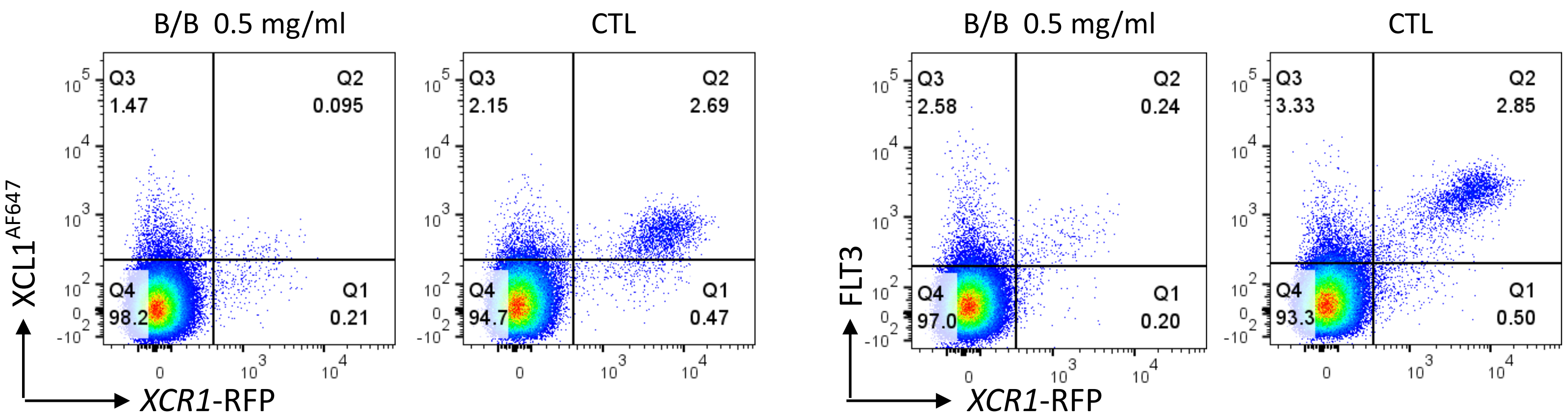

B

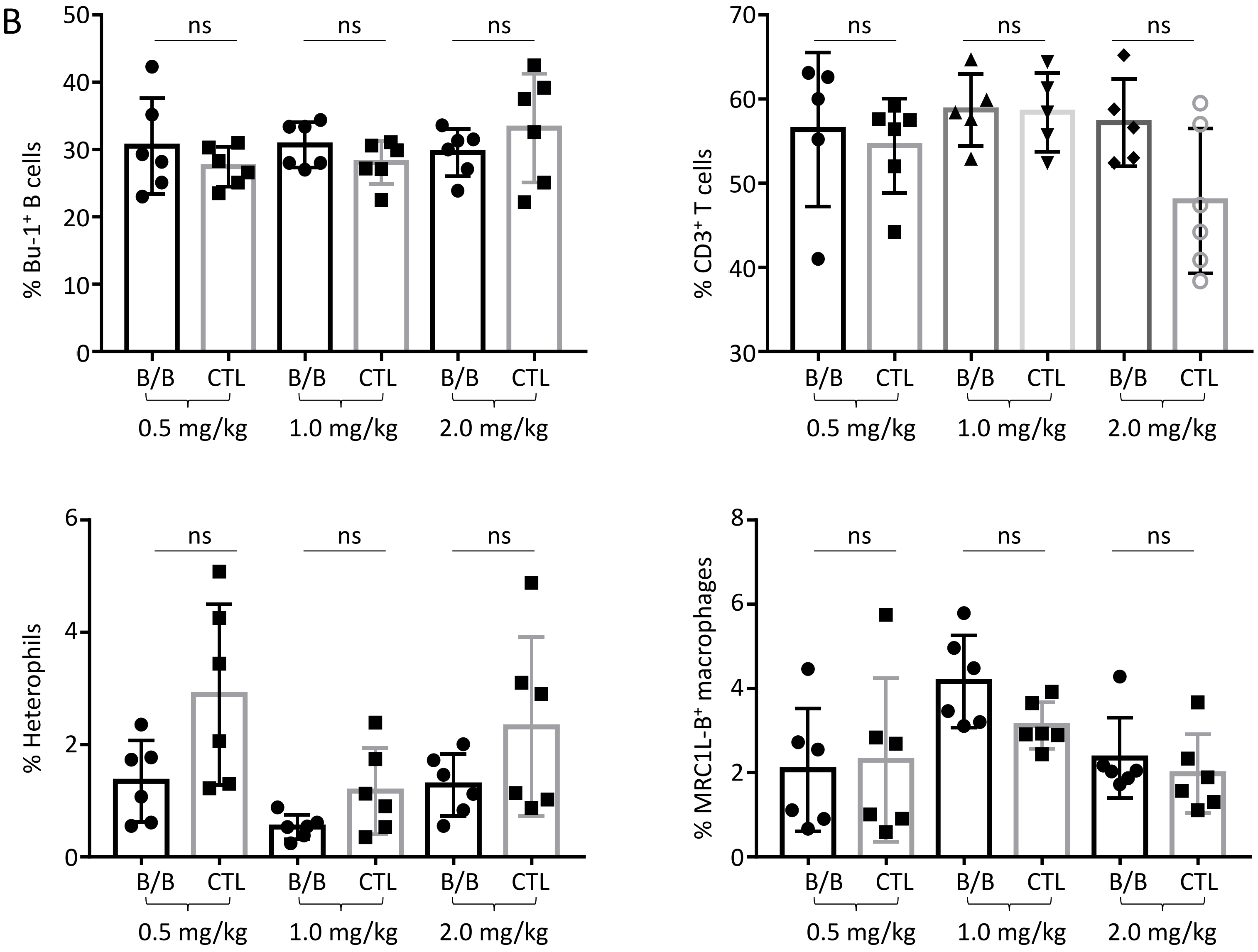

Supplementary Figure 10

A

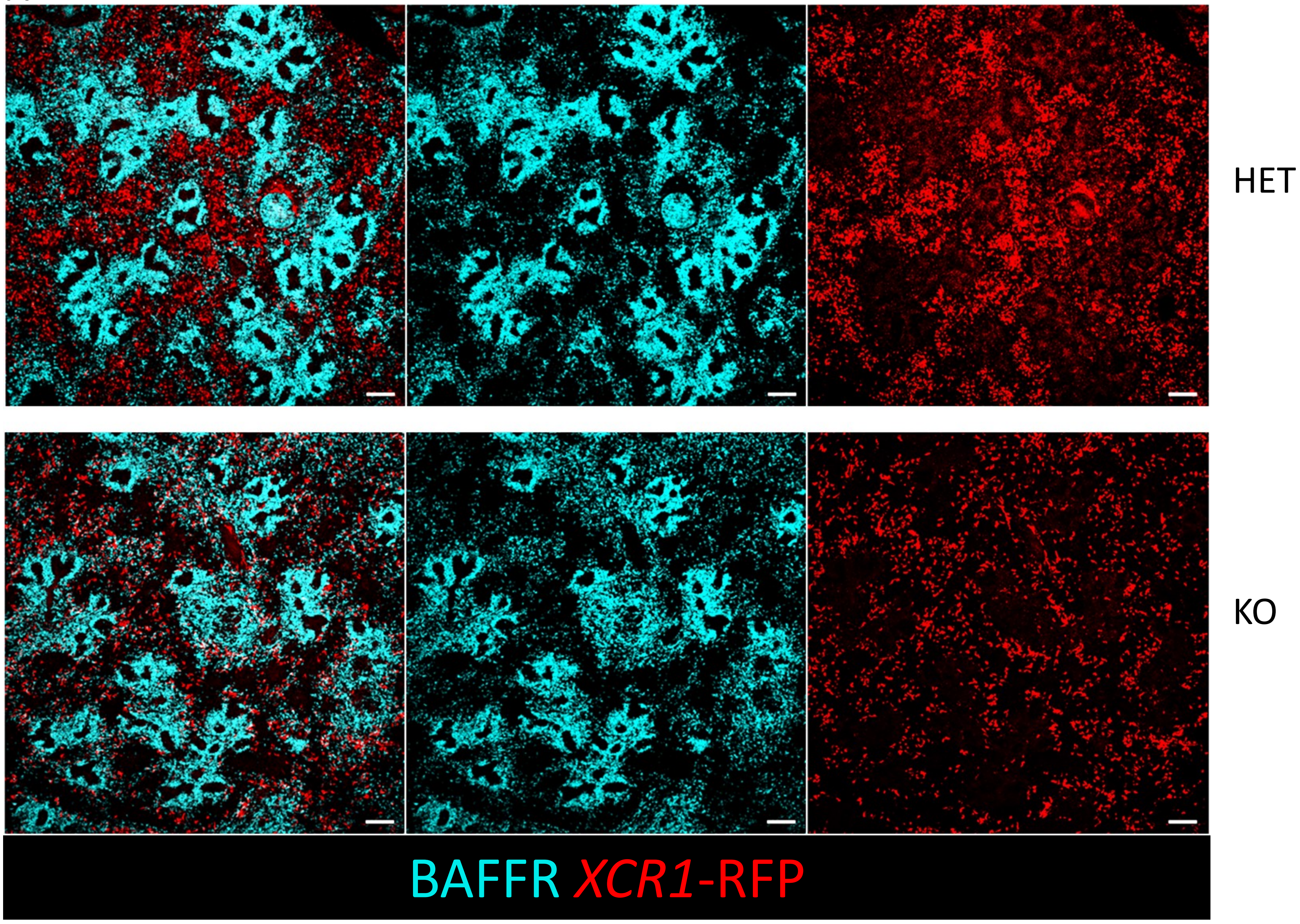

B

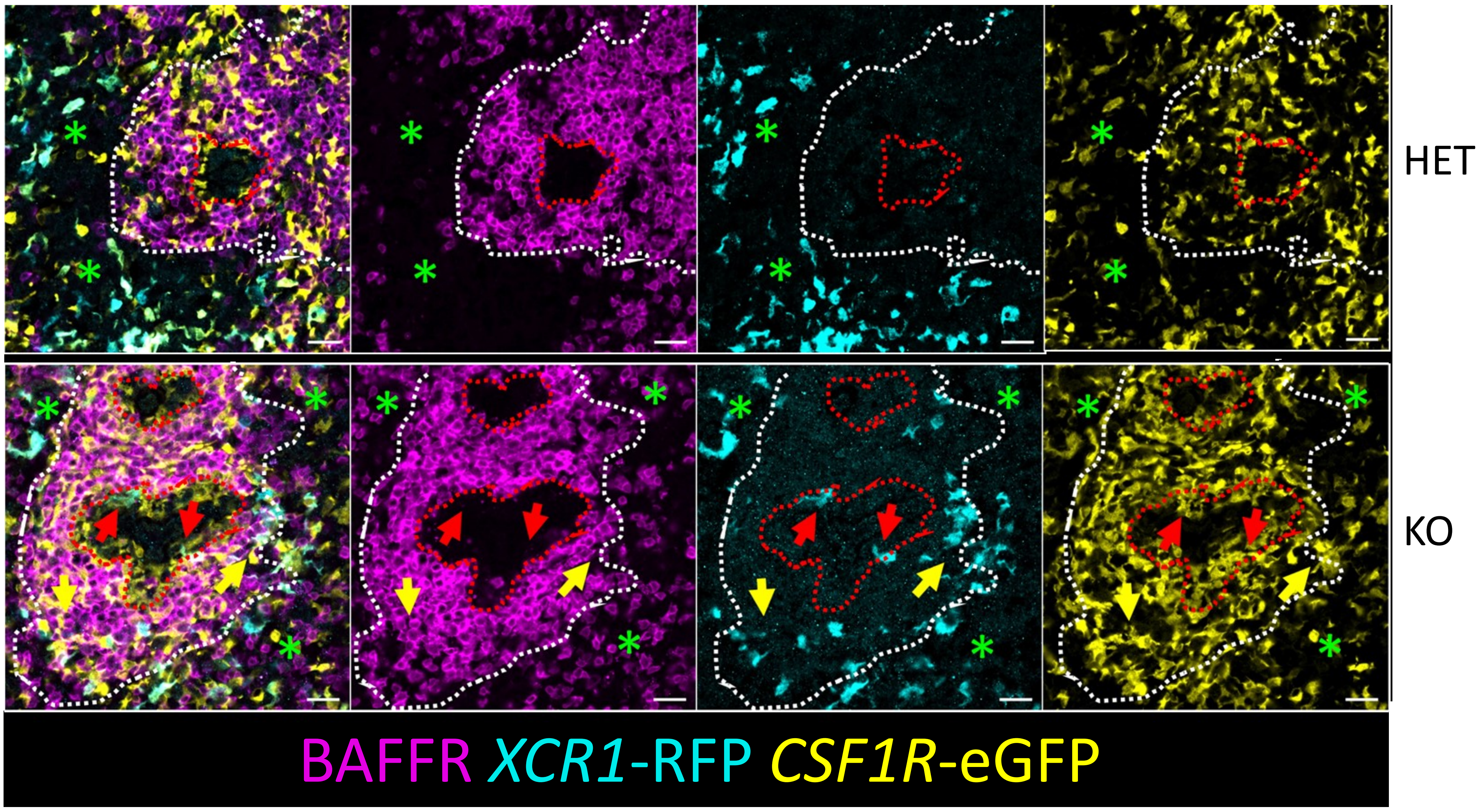

Supplementary Figure 11

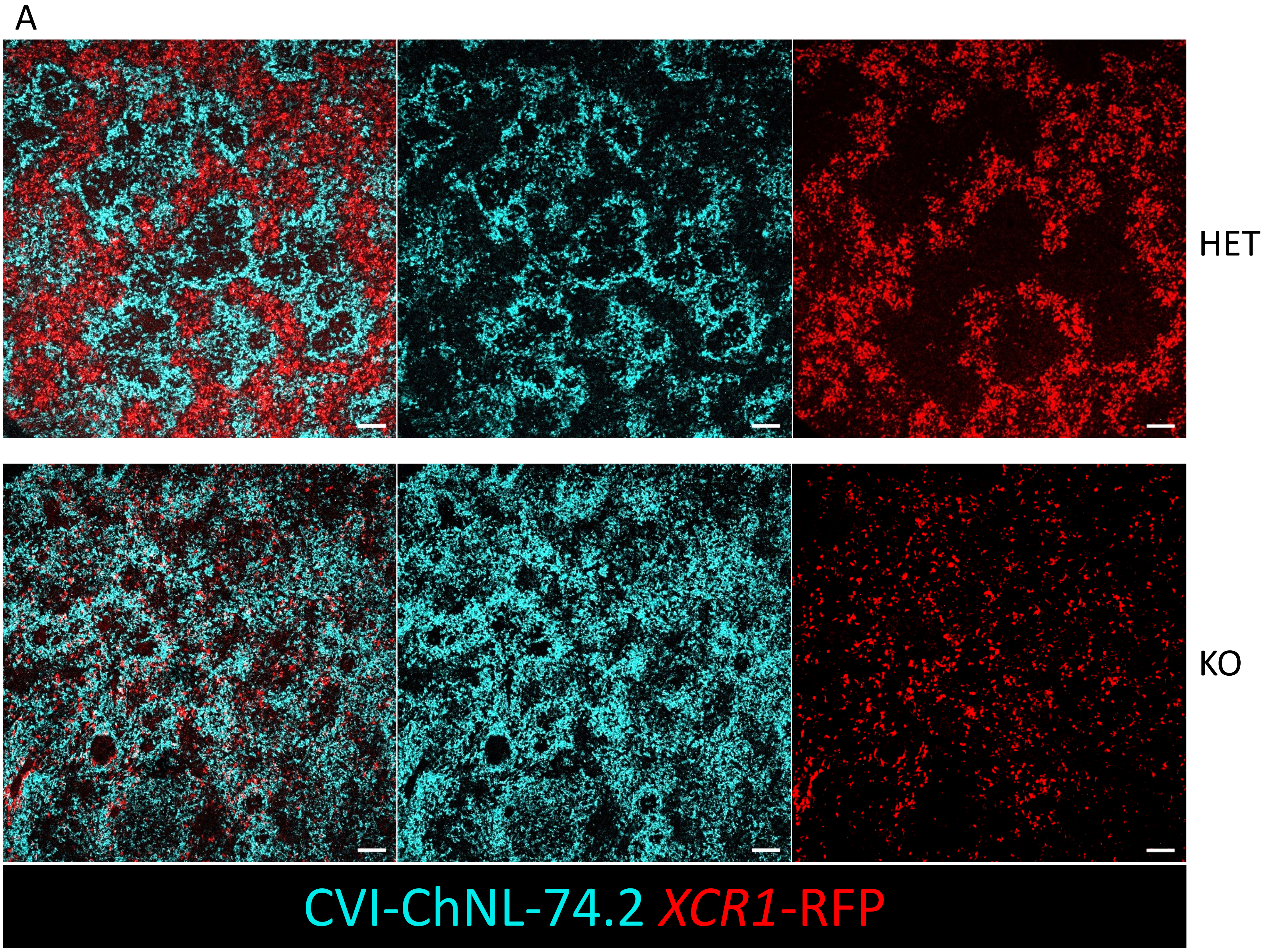

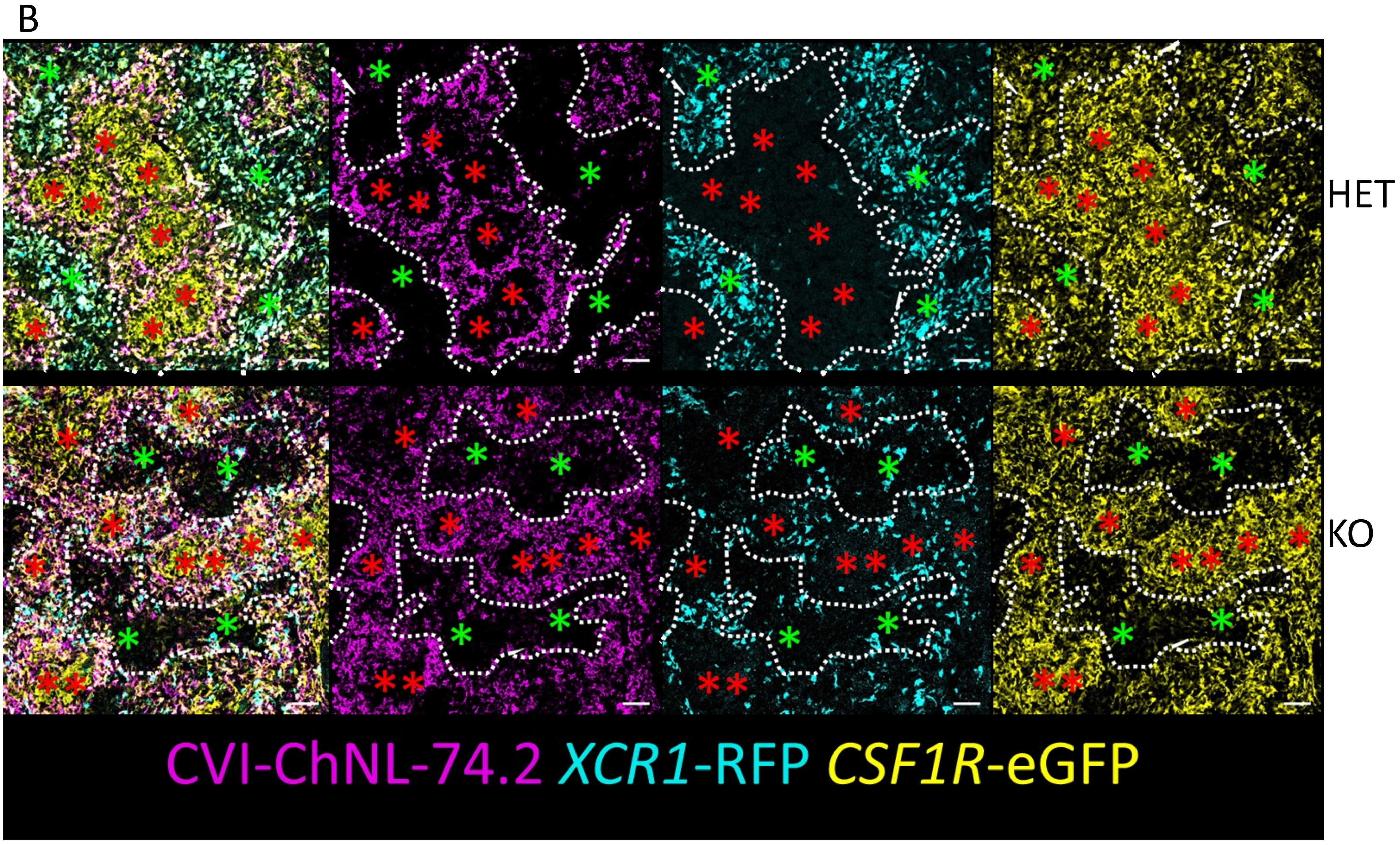

Supplement: Supplementary Figure 1 — PCR screening of edited PGC and gene edited chickens. [file DataSheet_1.pdf]
